# Grain Boundary‐Rich Copper Nanocatalysts Generated from Metal‐Organic Framework Nanoparticles for CO_2_‐to‐C_2+_ Electroconversion

**DOI:** 10.1002/advs.202207187

**Published:** 2023-01-22

**Authors:** Sungjoo Kim, Dongwoo Shin, Jonghyeok Park, Jong‐Yeong Jung, Hyunjoon Song

**Affiliations:** ^1^ Department of Chemistry Korea Advanced Institute of Science and Technology Daejeon 34141 Republic of Korea; ^2^ Department of Chemistry Seoul National University Seoul 08826 Republic of Korea

**Keywords:** copper oxide, electrochemical carbon dioxide reduction, grain boundaries, metal‐organic frameworks, morphology control, nanocatalysts

## Abstract

Due to severe contemporary energy issues, generating C_2+_ products from electrochemical carbon dioxide reduction reactions (eCO_2_RRs) gains much interest. It is known that the catalyst morphology and active surface structures are critical for product distributions and current densities. Herein, a synthetic protocol of nanoparticle morphology on copper metal‐organic frameworks (n‐Cu MOFs) is developed by adjusting growth kinetics with termination ligands. Nanoscale copper oxide aggregates composed of small particulates are yielded via calcining the Cu‐MOF nanoparticles at a specific temperature. The resulting nanosized MOF‐derived catalyst (n‐MDC) exhibits Faradaic efficiencies toward ethylene and C_2+_ products of 63% and 81% at −1.01 V versus reversible hydrogen electrode (RHE) in neutral electrolytes. The catalyst also shows prolonged stability for up to 10 h. A partial current density toward C_2+_ products is significantly boosted to −255 mA cm^−2^ in an alkaline flow cell system. Comprehensive analyses reveal that the nanoparticle morphology of pristine Cu MOFs induces homogeneous decomposition of organic frameworks at a lower calcination temperature. It leads to evolving grain boundaries in a high density and preventing severe agglomeration of copper domains, the primary factors for improving eCO_2_RR activity toward C_2+_ production.

## Graphical abstract

A nanoparticle morphology of copper metal‐organic frameworks is induced by adjusting growth kinetics. Calcination at a specific temperature develops dense grain boundaries in copper domains, resulting in superior catalytic performances on C_2+_ production from the electroreduction of CO_2_.

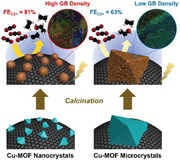

## 1 Introduction

Electrochemical carbon dioxide reduction reaction (eCO_2_RR) has become a center of topics in energy‐related issues because it can complete a closed loop of carbon utilization and production, combined with renewable energy sources.^[^ ^1^ ^]^ In particular, there has been a desire to find an efficient way to convert CO_2_ into C_2+_ products, such as ethylene, ethanol, and propanol, since these products have high energy density per molecule and can be utilized as essential resources in versatile chemical processes.^[^ ^2^ ^]^ Generally, copper is a well‐known CO_2_ reduction catalyst for producing C_2+_ products due to its moderate binding energy against *CO intermediates suitable for invoking dimerization processes. However, the low selectivity toward the desired C_2+_ becomes a big obstacle for eCO_2_RR.^[^ ^3^ ^]^

Several reports demonstrated that oxide‐derived copper catalysts exhibited improved C_2+_ product selectivity.^[^ ^4^, ^5^ ^]^ Then, critical factors, including morphology,^[^ ^6^, ^7^, ^8^ ^]^ oxidation states,^[^ ^9^, ^10^ ^]^ subsurface oxygen,^[^ ^11^ ^]^ vacancies,^[^ ^12^ ^]^ and hydrophobicity,^[^ ^13^ ^]^ have widely been investigated. Yeo et al. observed a strong correlation between the crystallite sizes and ethylene selectivity, where smaller ones showed higher Faradaic efficiency (FE) toward ethylene production, up to 43%.^[^ ^14^ ^]^ Buonsanti et al. showed facet‐dependent selectivity of copper catalysts with high current densities in a gas‐fed flow cell.^[^ ^15^ ^]^ Copper oxide nanoparticles bearing {100} and {111} surface facets exhibited the maximum FE of 59% for ethylene production.^[^ ^7^ ^]^ Hwang et al. and Song et al. observed the fragmentation of the catalysts into tiny domains in a few nm diameters during eCO_2_RR, resulting in highly enhanced ethylene selectivity.^[^ ^16^ ^]^ Generating abundant grain boundaries in copper nanoparticles also promoted multicarbon selectivity in eCO_2_RR due to increased binding strength upon *CO at the grain boundaries.^[^ ^17^, ^18^ ^]^

Recently, metal‐organic frameworks (MOFs) have been of interest because of their atomically dispersed metal sites and facile composition tunability.^[^ ^19^ ^]^ Thermal treatment decomposed organic linkers of the Cu‐MOFs and generated either copper oxide clusters or nanoparticles, which were expected to be active species for eCO_2_RR.^[^ ^20^, ^21^ ^]^ However, the catalysts showed C_2+_ selectivity inferior to other copper oxide nanostructures. Despite the atomic‐level control plausible in original MOF structures, crystals produced through standard solvothermal processes are larger than micrometers.^[^ ^22^ ^]^ It would be less favorable for suppressing agglomerations of catalytically active domains after the thermal decomposition. If the crystal size of the MOFs drops into a nanometer range, heat flux can be evenly applied to each crystallite, resulting in the formation of uniform catalyst structures.^[^ ^23^ ^]^ Although some synthetic techniques were introduced for specific materials, the catalytic applications of nanosized MOFs were rarely reported.^[^ ^24^ ^]^

In the present work, we prepared copper oxide nanocrystals by controlled thermal decomposition of Cu‐MOF nanoparticles. The crystal size of Cu‐MOFs was diminished to a nanometer scale by a seed‐mediated rapid growth in the presence of termination ligands. The thermal treatment induced the formation of copper oxide nanoparticles bearing small domains less than 10 nm. Spectroscopic and microscopic analyses revealed numerous grain boundaries generated in the catalyst particles. Consequently, the catalyst exhibited 63.1% of the FE toward ethylene and 80.9% toward total C_2+_ products at −1.01 V versus reversible hydrogen electrode (RHE) under neutral conditions in eCO_2_RR, far superior to the catalysts derived from microparticles. The catalyst also showed prolonged durability of 10 h. The catalyst was also employed in a flow cell system with an alkaline electrolyte; the partial current density was attainable up to −255 mA cm^−2^. These results indicate that a pre‐treatment or activation stage just before the reaction is critical in generating catalytically active domain structures for eCO_2_RR. The nanosized MOFs generated grain boundary‐rich copper domains and induced a large enhancement of C_2+_ production.

## 2 Results

### 2.1 Morphology Control of Cu‐MOFs into Nanoparticles

**Figure 1:**
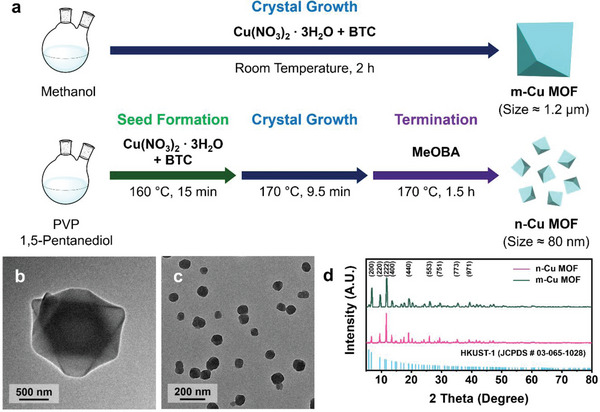
Synthesis of MOFs as catalyst precursors. a) Synthetic protocols of m‐ and n‐Cu MOFs. TEM images of b) m‐ and c) n‐Cu MOFs. d) XRD spectra of m‐ (green) and n‐Cu MOFs (pink).

We tried to generate active copper catalysts with small domain sizes having many grain boundaries. The catalysts should be highly dispersed on conductive carbon supports. In this regard, Cu‐MOFs were the best candidate among various precursor materials due to their high atomic‐level tunability. HKUST‐1 was our choice of material since it forms a stable crystal structure with a uniform distribution of metal centers linked by simple organic ligands.^[^ ^25^ ^]^ The mixture of copper nitrates and 1,3,5‐benzenetricarboxylic acid (BTC) in methanol at room temperature generally yielded large microcrystals (m‐Cu MOF) with an octahedral morphology (**Figure** **1**a,b).^[^ ^26^ ^]^ The average size of m‐Cu MOF was estimated to be 1.2 ± 0.3 µm from transmission electron microscopy (TEM) (Figure 1b) and scanning electron microscopy (SEM) images (Figure S1, Supporting Information).

We expected that precursor morphology would significantly affect the active catalyst structure generated during an activation process. Hence, we pursued synthesizing discrete nanoparticles composed of Cu‐MOFs. In general, a rapid nucleation rate followed by slow growth can reduce the crystal size.^[^ ^27^ ^]^ Unlike the microcrystal formation through a single growth step, we divided the synthesis into three subsequent steps—seed formation, crystal growth, and termination (Figure 1a). First, small crystallites were synthesized by adding a small amount of the precursor solution into 1,5‐pentanediol at a high temperature.^[^ ^28^ ^]^ This polyol process at a high thermal flux enabled rapid nucleation.^[^ ^29^ ^]^ Second, the particles were grown by dropwise addition of the precursors in the presence of poly(vinylpyrrolidone) (PVP). It prevented further nucleation and led to a narrow size distribution.^[^ ^30^ ^]^ Lastly, a termination ligand, 4‐methoxybenzoic acid (MeOBA), was introduced into the reaction mixture. This ligand has a carboxylate functionality competing with BTC to coordinate Cu^2+^ but terminated the extended growth due to the lack of a chelating site.^[^ ^31^ ^]^ After the synthesis, the resulting morphology of Cu‐MOF was obtained as spherical particles with an average diameter of 80 ± 13 nm (n‐Cu MOF, Figure 1c and Figure S2, Supporting Information). The particle size dramatically decreased by 14 times through this multistep process. Although the crystal size was largely distinct, the chemical compositions of m‐ and n‐Cu MOFs were identical. The powder X‐ray diffraction (XRD) indicated that the peak patterns of both samples matched HKUST‐1 (JCPDS No. 03‐065‐1028, Figure 1d). The extended X‐ray fine structures (EXAFS) of m‐ and n‐Cu MOFs also corresponded to HKUST‐1 (Figure S3 and Table S1, Supporting Information).^[^ ^32^ ^]^

### 2.2 Preparation of the MDC Catalysts by Thermal Treatment

**Figure 2:**
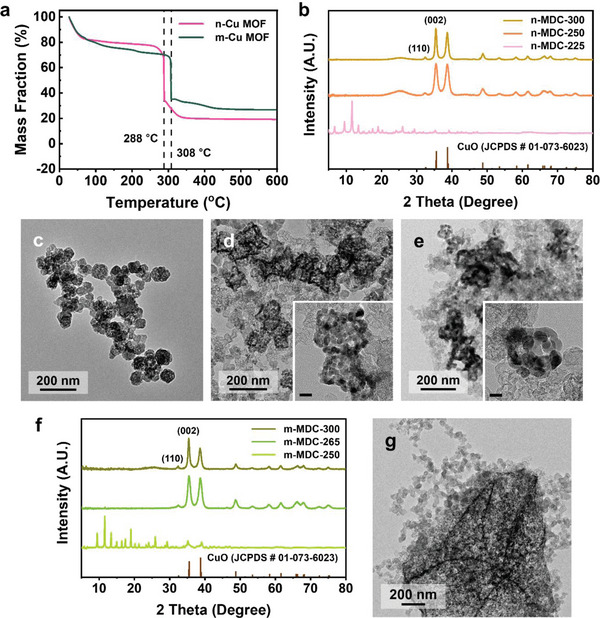
Generation of MDCs by thermal treatment. a) TGA profiles of m‐ and n‐Cu MOFs on Ketjen black. b) XRD spectra and TEM images of n‐MDC treated at c) 225, d) 250, and e) 300 °C. The scale bars of inset images represent 20 nm. f) XRD spectra of m‐MDC calcined at 250, 265, and 300 °C. g) TEM image of m‐MDC treated at 265 °C.

We attempted to determine the optimal thermal treatment conditions to prepare copper oxide catalysts. The Cu‐MOFs were cast on a conductive carbon support, Ketjen black, and thermogravimetric analysis (TGA) was performed. For n‐Cu MOF, the initial drop in TGA was caused by water desorption. Then, the abrupt declination of ≈50 wt % was observed at 288 °C, where the organic moieties were decomposed (**Figure** **2**a, pink line).^[^ ^33^ ^]^ Based on this result, we selected three representative temperatures for calcination, 225 °C, 250 °C, and 300 °C; 225 °C and 300 °C were temperatures before and after the complete decomposition of organic ligands. 250 °C was the threshold point where the decomposition began. The n‐Cu MOF on the support was calcined at these fixed temperatures for 3 h. The resulting materials were referred to as n‐MDC‐225, 250, and 300, meaning nanosized MOF‐derived copper catalysts treated at the designated temperatures. The chemical composition and morphology of n‐MDCs were identified by XRD and TEM. The XRD pattern of n‐MDC‐225 was identical to HKUST‐1 due to insufficient thermal energy applied for degrading MOF structures (Figure 2b). The TEM image also verified an unchanged morphology of n‐Cu MOF (Figure 2c). On the other hand, all diffractions of the MOF structure disappeared, and new intense peaks assignable to CuO (JCPDS No. 01‐073‐6023) appeared in n‐MDC‐250 and 300. It indicated that n‐Cu MOF was successfully transformed into CuO by the thermal treatment over 250 °C.

However, n‐MDC‐250 and 300 showed a significant difference in their morphology. In n‐MDC‐250, the extrinsic morphology maintained its original shape with similar sizes of ≈80 nm. Each particle was composed of small CuO particulates with an average diameter of 8.6 ± 1.6 nm, constituting a superparticle morphology (Figure 2d and inset). From Scherrer's equation using prominent (110) and (200) XRD peaks, the average single‐crystalline domain size was estimated to be 9.3 nm, in good agreement with the TEM image (Table S2, Supporting Information). On the contrary, neighboring CuO particles were severely agglomerated in n‐MDC‐300, forming irregular hollow aggregates (Figure 2e and inset). The domain size was 14.5 nm, estimated from the TEM image and XRD peaks. Halder‐Wagner's plots also verified the crystalline domain sizes from the XRD peaks for n‐MDC‐250 (9.1 nm) and n‐MDC‐300 (14.2 nm) (Figure S4 and Table S3, Supporting Information). It revealed that the decomposition of organic ligands and reorganization of metal ions into CuO were highly susceptible to the calcination temperature. At the threshold temperature of 250 °C, the CuO domains were begun to generate by ligand decomposition. Still, the domain growth and agglomeration with neighboring particles did not occur due to insufficient thermal energy supply. There was also a possibility that the organic moieties partially remained and prevented rapid growth of the CuO domains.^[^ ^34^ ^]^ At 300 °C, the organic ligands were removed entirely, and the excess thermal energy was enough to agglomerate metal domains to make irregular aggregates.

For m‐Cu MOF, the mass fraction slowly decreased by increasing the temperature after the initial drop before 100 °C (Figure 2a, green line). It may be caused by retarded diffusion of adsorbates from the deep sides of microcrystals. Then, an abrupt drop occurred at 308 °C, shifted by 20 °C to n‐Cu MOF. Consequently, the XRD pattern of m‐MDC‐250, microsized MOF‐derived copper catalyst treated at 250 °C, was unchanged from the original HKUST‐1, while the n‐MDC‐250 changed to gain characteristic CuO peaks (Figure 2f). The MOF microcrystals with confined edges were retained after the thermal treatment (Figure S5a, Supporting Information). The CuO peak patterns were clearly identified in m‐MDC‐265 and 300, revealing that the threshold temperature of organic ligand decomposition in m‐MDC was about 265 °C. Microsized aggregates composed of CuO nanoparticles were observed in m‐MDC‐265 (Figure 2g and Figure S5b, Supporting Information). In m‐MDC‐300, big irregular agglomerates were formed on the carbon support (Figure S5c, Supporting Information).

The decomposition temperature gap in n‐ and m‐MDCs was attributed to the difference in thermal conduction behaviors. Due to the small particle size, thermal energy could effectively transfer to all parts simultaneously in n‐Cu MOF. The MOF structure shrank from the original morphology by decomposing organic ligands, and the resulting CuO domains were formed within a confined volume of the pristine n‐MDC. In contrast, inefficient thermal transfer to the deep sides of m‐Cu MOF made the decomposition of organic moieties difficult and the decomposition temperature higher than in n‐Cu MOF. By the high‐temperature treatment, the excess thermal energy and a massive volume of original microcrystals caused severe aggregation of CuO nanoparticles in m‐MDC. We also checked the interference from bare Ketjen black. No sudden drops of its mass for up to 600 °C signified that TGA profile changes originated from the catalyst precursor decomposition (Figure S6, Supporting Information).

### 2.3 eCO_2_RR Study in the H‐Cell

The study of eCO_2_RR using n‐ and m‐MDC families was performed in a three‐electrode H‐cell system in 0.1 m KHCO_3_ aqueous solution (Figure S7, Supporting Information). The loading amount of copper on the carbon support was fixed to 30 wt % for all samples based on inductively coupled plasma–optical emission spectroscopy (ICP–OES) (Table S4, Supporting Information). Both gaseous and liquid products were quantified by gas chromatography and proton nuclear magnetic resonance (^1^H‐NMR) spectroscopy (Figure S8, Supporting Information). The catalysts were activated before the reaction study by applying −1.35 V versus RHE for 10 min. The linear sweep voltammetry (LSV) confirmed the reduction of CuO and Cu_2_O to Cu after the activation (Figure S9, Supporting Information).

**Figure 3:**
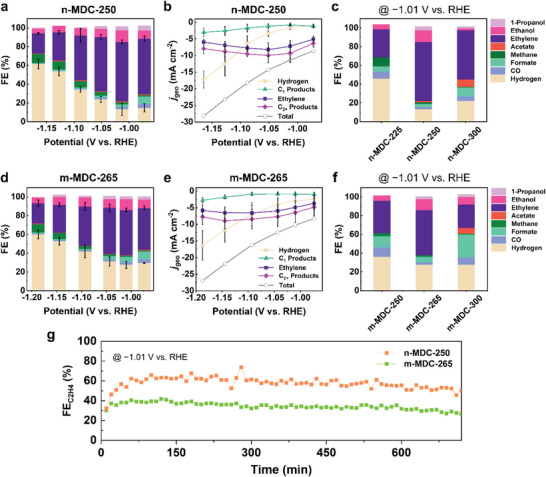
eCO_2_RR under a neutral environment. a) FE and b) geometric current density versus applied potential using n‐MDC‐250. c) FEs at −1.01 V versus RHE using n‐MDC‐225, 250, and 300. d) FE and e) geometric current density versus applied potentials using m‐MDC‐265. f) FEs at −1.01 V versus RHE using m‐MDC‐250, 265, and 300. g) Temporal FE_C2H4_ changes of n‐MDC‐250 and m‐MDC‐265.

**Figure** **3**a presents the FE of the products in eCO_2_RR using n‐MDC‐250. At −1.01 V versus RHE, the FE toward ethylene production (FE_C2H4_) reached a maximum of 63.1 ± 1.9%, whereas the FE toward hydrogen production (FE_H2_) was limited to 13.4 ± 6.5% (Table S5, Supporting Information). Gaseous C_1_ products, including CO and methane, were negligible. Among liquid products, ethanol was the primary product with an FE of 12.5%, and the sum of the other products, including 1‐propanol, formate, and acetate, was 8.4%. Consequently, the total FE of C_2+_ products (FE_C2+_) reached 80.9% at this optimized potential. By increasing the applied potential, the FE_H2_ gradually increased, while those of FE_C2H4_ and FE_C2+_ decreased.

The geometrical current density (*j*_geo_) versus applied potential was plotted in eCO_2_RR using n‐MDC‐250 (Figure 3b). At −1.01 V versus RHE, the total *j*_geo_ was −11.4 mA cm^−2^, and the *j*_geo_ of ethylene was −7.2 ± 1.7 mA cm^−2^. The mass activities toward ethylene and total C_2+_ products were estimated to be 103 mA mg^−1^ and 133 mA mg^−1^, respectively (Figure S10, Supporting Information). The current density of total C_2+_ products rose to −10.0 mA cm^−2^, then gradually decayed as the applied potential was increased. This behavior could be ascribed to the mass transport limitation of CO_2_, caused by poor aqueous solubility of CO_2_ and local pH increase near the electrode surface.^[^ ^35^ ^]^ The product analyses were also carried out for n‐MDC‐225 and 300 (Figure 3c and Table S6, Supporting Information). n‐MDC‐225 exhibited a low selectivity toward C_2+_ products, 29.6% for FE_C2H4_ and 34.8% for FE_C2+_. Instead, the FEs toward hydrogen (45.9%) and methane (9.8%) were distinctive compared to other samples. n‐MDC‐300 presented similar product distributions but less selectivity toward C_2+_ products than n‐MDC‐250, 52.6% for FE_C2H4_ and 64.3% for FE_C2+_. The massive difference in n‐MDC‐225 compared to n‐MDC‐250 and 300 indicated that forming copper/copper oxide particles was critical for producing multicarbon products. For the microsized aggregates, m‐MDC‐265, the FE_C2H4_ and FE_C2+_ reached 48.3 ± 1.9% and 62.9% at −1.01 V versus RHE, respectively (Figure 3d and Table S7, Supporting Information). At the optimized potential, the total *j*_geo_ and *j*_geo_ toward ethylene were −10.3 mA cm^−2^ and −4.9 ± 2.1 mA cm^−2^ (Figure 3e). The mass activities toward ethylene and total C_2+_ products were estimated to be 76 mA mg^−1^ and 101 mA mg^−1^, inferior to n‐MDC‐250 (Figure S10, Supporting Information). Similarly to n‐MDC‐250, the parabolic behavior in *j*_geo_ of C_2+_ was also observed in m‐MDC‐265, where the current density of C_2+_ products was limited to −9.6 mA cm^−2^. m‐MDC‐250 and 300 exhibited 40.3% and 42.1% for FE_C2+_, indicating the optimal structure of m‐MDC‐265 (Figure 3f and Table S8, Supporting information).

These MDC families were far better than commercial CuO nanoparticles (80 nm in diameter) for eCO_2_RR under identical neutral reaction conditions. The CuO catalyst cast on Ketjen black exhibited the FE_C2H4_ and FE_C2+_ of 36.5% and 46.8% at −1.01 V versus RHE with low geometric current densities (Figure S11, Supporting Information).

The long‐term durability for eCO_2_RR at −1.01 V versus RHE was tested for our best samples, n‐MDC‐250 and m‐MDC‐265 (Figure 3g). n‐MDC‐250 maintained the FE_C2H4_ of more than 60% up to 5 h and still showed a stable feature over 55% at 10 h. On the other hand, m‐MDC‐265 performed the FE_C2H4_ of 40% under this condition, and the selectivity gradually declined to 30% after 10 h operation. It revealed that n‐MDC had superior reaction properties to m‐MDC in many aspects, including reaction activity, selectivity, and durability for eCO_2_RR.

### 2.4 eCO_2_RR Study in the Gas Diffusion Flow Cell

**Figure 4:**
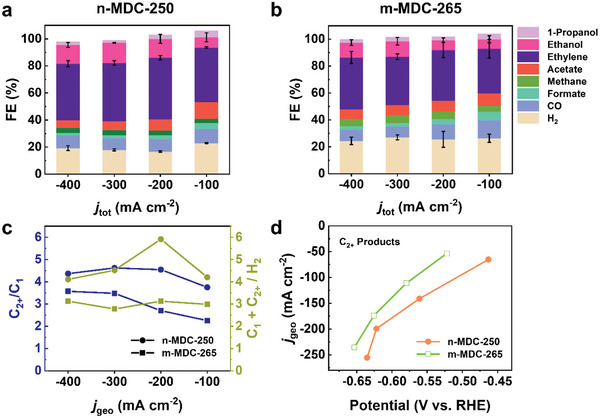
eCO_2_RR in alkaline flow cells. FE versus total geometric current density using a) n‐MDC‐250 and b) m‐MDC‐265. c) The ratios of C_2+_/C_1_ (blue) and CO_2_RR/HER (olive) products versus total geometric current density. d) Partial geometric current density of C_2+_ products versus the applied *iR*‐corrected potential for n‐MDC‐250 and m‐MDC‐265.

Due to the slow mass transport of CO_2_ in an aqueous medium, the geometric current density was severely limited in the H‐cell configuration. Instead, a flow cell system based on gas diffusion electrodes (GDEs) has been introduced to achieve high production rates.^[^ ^15^ ^]^ We fabricated a GDE cell with a gas‐fed flow system and performed eCO_2_RR for n‐MDC‐250 and m‐MDC‐265 under alkaline conditions (Figure S12, Supporting Information). The catalyst samples thermally treated at specific temperatures were deposited on carbon paper. Multiple spectroscopic and microscopic characterizations represented that the MDCs were successfully generated and loaded on the gas diffusion layers (Figures S13 and S14, Supporting Information). Under a continuous flow of 1.0 m KOH electrolyte, the product FEs was not significantly changed to the total geometric current density. For n‐MDC‐250, C_2+_ products majorly yielded close to the FE of 70%: for instance, at −200 mA cm^−2^, the FE_C2+_ was the maximum of 70.6%, including the FEs for ethylene, ethanol, acetate, and 1‐propanol of 45.6%, 13.9%, 8.2%, and 3.0%, respectively (**Figure** **4**a and Table S9, Supporting Information).

It was noticeable that the formation of liquid products was primarily enhanced compared to those in the neutral environment. It may be ascribed to the inhibition of the ethylene production pathway amid high OH^−^ concentration.^[^ ^36^ ^]^ The FEs toward H_2_ and C_1_ products were also suppressed to 15.2% and 15.7%. For m‐MDC‐265, the FE_C2+_ was 55.4% at −200 mA cm^−2^, with the FEs toward ethylene and ethanol of 37.7% and 7.4%, respectively (Figure 4b and Table S10, Supporting Information).

The differences between the two samples were analyzed regarding relative product ratios and partial geometric current densities. The maximum C_2+_ versus C_1_ product ratio was 4.6 for n‐MDC‐250 but was 2.7–3.5 for m‐MDC‐265 at −200 and −300 mA cm^−2^ (Figure 4c). The total eCO_2_RR versus hydrogen evolution reaction (HER) product ratio was also significantly higher for n‐MDC‐250 (> 4.0) than for m‐MDC‐265 (< 3.6). The partial geometric current densities of the major products were plotted (Figure 4d). For n‐MDC‐250, *j*_geo_ of C_2+_ products was measured to be −255 mA cm^−2^ at −0.64 V versus RHE in the flow cell, which was boosted 26 times higher than in the H‐cell configuration. *j*_geo_ of C_2+_ products for m‐MDC‐265 was −236 mA cm^−2^ at −0.65 V versus RHE, a 8.1% smaller density at a higher overpotential. The mass activity toward the C_2+_ products was estimated to be 354 mA mg^−1^ at −0.56 V versus RHE for n‐MDC‐250, 1.5‐fold larger than 232 mA mg^−1^ for m‐MDC‐265 (Figure S15, Supporting Information). These eCO_2_RR studies indicated that the catalytic properties of n‐MDC were superior to m‐MDC regardless of reaction conditions (in neutral or alkaline electrolytes) and cell configurations (H‐cell or flow cell systems). The catalytic properties were stable for 2 h under the alkaline flow cell system, comparable to copper catalysts in the GDE flow cell configuration.^[^ ^15^ ^]^

The Cu‐coordinated MOFs have been utilized as a precursor suitable for generating copper oxide catalysts in eCO_2_RR (Table S11, Supporting Information).^[^ ^20^, ^21^, ^37^, ^38^ ^]^ Quan et al. pyrolyzed Cu‐MOF microcrystals, yielding copper oxide porous aggregates. The catalyst exhibited a FE toward ethanol production of 35% at maximum under the neutral condition.^[^ ^20^ ^]^ Sargent et al. reported that controlled calcination induced Cu dimer distortion, generating Cu clusters with under‐coordinated sites. The resulting catalyst improved FE toward ethylene by up to 45% in a flow cell.^[^ ^37^ ^]^ Liang et al. thermally treated Cu‐MOFs and tracked the complete conversion into inorganic Cu_2_O/CuO aggregates. The catalyst showed a maximum FE_C2H4_ of 51% and FE_C2+_ of 70% at −1.58 V versus RHE.^[^ ^21^ ^]^ Among these experiments, n‐MDC‐250 exhibited superior ethylene and C_2+_ product selectivities and comparable current densities under the various reaction conditions, presumably due to the precise morphology control in nanoscale treated at the optimal temperature. Even compared to the oxide‐derived copper catalysts, n‐MDC‐250 showed comparable or better performances both in H‐ and flow‐cell configurations (Table S12, Supporting Information).^[^ ^5^, ^7^, ^8^, ^12^, ^13^, ^16^, ^39^, ^40^ ^]^

### 2.5 Structural and Compositional Analyses of the Active Catalyst Domains

**Figure 5:**
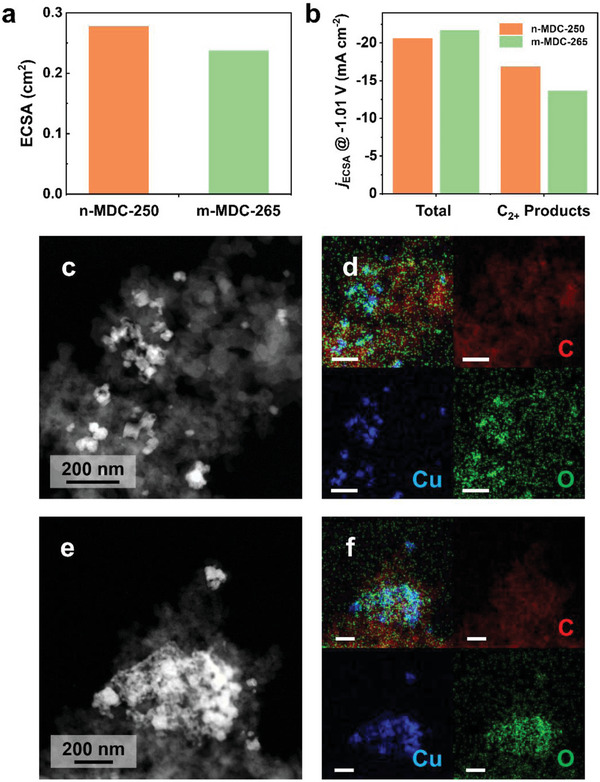
Surface areas and morphologies of the MDC catalysts. a) ECSAs and b) *j*_ECSA_s of total and C_2+_ products using n‐MDC‐250 and m‐MDC‐265 at −1.01 V versus RHE. c) STEM‐HAADF and d) elemental mapping images of n‐MDC‐250 after the activation. e) STEM‐HAADF and f) elemental mapping images of m‐MDC‐265 after the activation. The Cu, O, and C elements are colored blue, green, and red. All scale bars represent 200 nm.

We examined the origin of this superior selectivity toward multicarbon products using n‐MDC compared to m‐MDC. As a bulk measurement, the electrochemically active surface area (ECSA) of the catalyst electrode was measured by Pb underpotential deposition on copper in cyclic voltammograms (Figure S16, Supporting Information). The Pb stripping peaks appeared at −0.35 V versus Ag/AgCl (3 m NaCl). The ECSA of n‐MDC‐250 after the activation was measured to be 0.28 cm^2^, while m‐MDC‐265 exhibited a less area of 0.24 cm^2^ (**Figure** **5**a), as expected from the irregular aggregates with large domains (Figure 2g). While the large surface area effect was removed, the resulting ECSA‐normalized current densities (*j*_ECSA_s) of the two samples were nearly identical (−21 mA cm^−2^) toward the total products at the optimized potential.^[^ ^41^ ^]^ However, *j*_ECSA_ toward C_2+_ products was still 20% higher in n‐MDC‐250 than in m‐MDC‐265, pointing out that the product selectivity did not only result from the large surface area but also the superior intrinsic activity of the nanosized aggregates (Figure 5b and Table S13, Supporting Information).^[^ ^41^ ^]^

The catalyst morphology after the activation was analyzed using scanning transmission electron microscopy‐high angular annular dark field (STEM‐HAADF) and scanning transmission electron microscopy‐energy dispersive spectroscopy (STEM‐EDS) elemental mapping techniques. After the electrochemical activation, the copper oxide particle aggregates with an average diameter of 50 ± 15 nm were dispersed on the carbon support in n‐MDC‐250 (Figure 5c,d). This morphology was hardly changed from the original structure of n‐MDC‐250 before the activation (Figure S17a,b, Supporting Information). On the other hand, the images of m‐MDC‐265 showed that the particles formed big irregular agglomerates after the activation (Figure 5e,f, and Figure S17c,d, Supporting Information). These morphology changes were clearly distinguished after eCO_2_RR for 12 h. Even after the prolonged reactions, the copper domains of n‐MDC‐250 were still dispersed well in the supports, maintaining an average particle size of 50 nm (Figure S18a, Supporting Information). In contrast, the m‐MDC‐265 underwent further agglomeration to make micrometer‐sized lumps (Figure S18b, Supporting Information), leading to the deterioration of reactivity.

**Figure 6:**
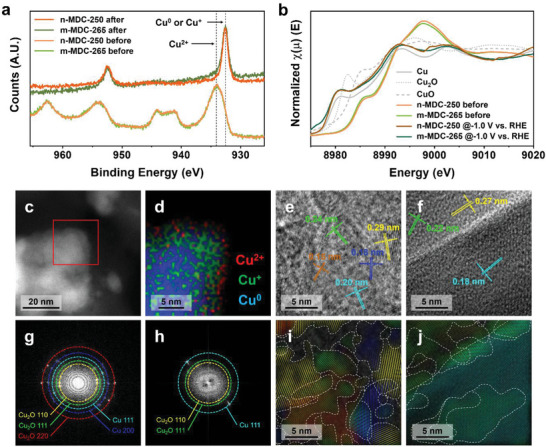
Chemical species and grain boundaries of the MDC catalysts. a) XPS spectra in Cu 2p and b) in situ Cu K‐edge XANES spectra for n‐MDC‐250 and m‐MDC‐265 before and after the activation at −1.0 V versus RHE with references. c) STEM‐ADF and d) STEM‐EELS mapping images of n‐MDC‐250. HRTEM images and corresponding FFT patterns of e,g) n‐MDC‐250 and f,h) m‐MDC‐265 after the activation. Inverse FFT mapping analysis of i) n‐MDC‐250 and j) m‐MDC‐265. The grain boundaries are indicated with white dashed lines.

Understanding the maximum selectivity toward ethylene and C_2+_ products needs more detailed information on active copper species and crystalline domain analysis. To enhance the selectivity toward multicarbon products, numerous factors, such as catalyst morphology, composition, crystal domains, and defects, may influence the catalyst performance. Tang et al. demonstrated that hierarchical porous structures could bolster the internal catalyst activities toward C_2_ products.^[^ ^42^ ^]^ Bao et al. substantiated that the residual Cu^+^ and adsorbed iodine species on the catalyst surface were responsible for enhanced C_2+_ production.^[^ ^43^ ^]^ Mo et al. proved that the nanoscale defects in the catalysts could promote C—C coupling for ethylene formation.^[^ ^44^ ^]^ In our MDC catalysts, we assessed the importance of active copper species. The surface oxidation states were measured by various X‐ray techniques. The X‐ray photoelectron spectra (XPS) confirmed the peaks at 934.1 eV, assignable to Cu^2+^ as major species (**Figure** **6**a, bottom). After the electrochemical activation of MDCs, the Cu^2+^ peaks completely disappeared in Cu 2p_3/2_ XPS, and the peaks at 932.5 eV corresponding to Cu^0^ or Cu^+^ were observed.^[^ ^45^ ^]^ Auger electron spectroscopy (AES) in the Cu LMM region also showed the peak maximum at 569.2 eV of MDCs before the activation was assigned to Cu^2+^, which moved to 570.3 eV assignable to Cu^+^ with a broad shoulder at 568.1 eV for Cu^0^ (Figure S19, Supporting Information).^[^ ^46^ ^]^ The X‐ray absorption near edge structures (XANES) were also taken to evaluate the average share of each copper species. Before the activation, XANES exhibited a distinct shoulder at 8985.9 eV and a major peak at 8997.9 eV in Cu K‐edges for MDCs, matching the CuO profile. After the activation, the shoulders appeared between Cu and Cu_2_O, indicative of Cu^0^ and Cu^+^ species present in both samples. (Figure S20 and Table S14, Supporting Information) We also performed in situ XANES for two MDCs before and after applying the potential of −1.0 V versus RHE (Figure 6b and Table S14, Supporting Information). The spectra were consistent with the ex situ experiments. In particular, the linear combination fitting demonstrated that Cu^2+^ was the major species before the activation, and it was completely reduced to Cu^0^ and Cu^+^ under the applied potential. Notably, Cu^+^ species were still present significantly (≈25%) for both samples. Still, all spectral features and changes before and after the activation were identical in n‐MDC‐250 and m‐MDC‐265. As well as the surface measurements, we observed the spatial distribution of copper species by scanning transmission electron microscopy‐annular dark field (STEM‐ADF) and scanning transmission electron microscope‐electron energy loss spectroscopy (STEM‐EELS) mapping images. In n‐MDC‐250, a small fraction of Cu^2+^ was presented only on the surface exposed outward, presumably generated by surface oxidation during the sample preparation.^[^ ^47^ ^]^ The particles were dominated by Cu^0^ and Cu^+^ species, where Cu^+^ was located within 5 nm from the surface (Figure 6c,d). Similar distributions were also observed in m‐MDC‐265. Although the aggregate size was huge, the Cu^0^ and Cu^+^ species were distributed over the aggregates with nearly negligible spots for Cu^2+^ (Figure S21, Supporting Information). All of these data were consistent in that Cu^0^ and Cu^+^ were dominant species in electrochemically activated MDCs, leading to CO_2_ conversion into C_2+_ products.^[^ ^9^, ^39^, ^48^ ^]^ Nonetheless, no considerable differences were observed in copper species after activating n‐MDC‐250 and m‐MDC‐265.

In addition, we considered the crystallinity of the catalyst materials. In the high‐resolution transmission electron microscopy (HRTEM) image of n‐MDC‐250 after the activation, tiny domains were identified in the small particulate. Each fringe image represented different orientations and spacings corresponding to individual facets (Figure 6e). The fast Fourier transform (FFT) patterns in the selected area had multiple dots, matching with (110), (111), and (220) planes of Cu_2_O or (111) and (200) planes of metallic Cu (Figure 6g). For m‐MDC‐265, a small number of lattice fringe images with distinct orientations were observed in the HRTEM image, and the corresponding FFT patterns represented only low index planes of Cu_2_O and Cu (Figure 6f,h). For detailed analysis, inverse FFT mapping was conducted with coloring different lattice fringes in Figure 6e,f.^[^ ^49^ ^]^ n‐MDC‐250 exhibited severe fragmentation of the single‐crystalline domains with extended grain boundaries (Figure 6i), whereas m‐MDC‐265 had relatively large domains (Figure 6j). Based on the acquired inverse FFT mapping images (Figure 6i,j), the grain boundary densities of the two samples were assessed by normalizing the total length of grain boundaries within the imaged region into the area of the analyzed region. The grain boundary density was estimated to be 794 µm^−1^ in n‐MDC‐250, 50% higher than 529 µm^−1^ in m‐MDC‐265 (Figure S22, Supporting Information). Detailed chemical environments of MDC samples were able to be assessed by EXAFS fitting (Figure S23, Supporting Information). The structural parameters of both samples before the activation were similar to CuO (Table S15, Supporting Information). After the electrochemical activation, the peaks assignable to CuO disappeared, while the Cu—Cu for Cu^0^ and Cu—O peaks for Cu_2_O became eminent. The change in peak intensities and coordination numbers were parallel with the observations made from other spectroscopic data (Table S16, Supporting Information).^[^ ^50^ ^]^ Debye‐Waller factors (*σ* ^2^) of n‐MDC‐250 were significantly higher than m‐MDC‐265 in the Cu—Cu pathways, envisioning more crystal structure deviations, such as defect sites and grain boundaries, were densely developed in n‐MDC‐250 during the activation.^[^ ^51^ ^]^ As mentioned, the nanosized aggregates had identical chemical composition and species to the microsized ones. Still, the multiple characterization results indicated that the nanoaggregates had a large grain boundary density with small domains, primarily causing the maximum selectivity toward ethylene and C_2+_ products in eCO_2_RR. This corroboration is in line with the other experiments proving the grain boundary as a critical factor for high activity in nanostructured copper oxides. Since Kanan et al. proposed grain boundaries as promoters for CO reduction, theoretical and empirical studies evidenced that dense grain boundaries or interfaces increased C_2+_ selectivity in eCO_2_RR.^[^ ^17^, ^18^, ^52^, ^53^, ^54^ ^]^ Gong et al. proved that the CO intermediates were adsorbed more strongly in the grain boundary‐rich catalyst surface.^[^ ^18^ ^]^ Han et al. suggested that high C_2+_ selectivities were facilitated by grain boundary networks and exposed high‐index facets in fragmented copper films other than specific Cu oxidation states.^[^ ^53^ ^]^ The existence of copper domains with grain boundary‐rich characters induced much strong adsorption of CO intermediates on the surface. It provided sufficient retention time of *CO to undergo CO dimerization through *OCCO intermediate species.^[^ ^54^ ^]^ It also suppressed the competitive hydrogen adsorption, following the proton‐coupled pathway to yield C_1_ products. As a result, C_2+_ product formation was distinctively enhanced in the grain boundary‐rich structure. Our study also proved that the high grain boundary density was directly associated with the FE toward ethylene and C_2+_ products instead of specific copper species and oxidation states.

## 3 Conclusion

In summary, we successfully tuned the morphology of Cu‐MOFs into nanoparticles with 80 nm diameters via a seed‐mediated solvothermal polyol process. This nanoscale morphology rendered the calcination temperature lower due to facile thermal conduction over the materials. Severe agglomeration was suppressed by a relatively mild thermal treatment to form small copper oxide domains with dense grain boundaries in the catalyst (n‐MDC‐250). The catalyst dispersion on the carbon support was also maintained during the reaction. The resulting MOF‐based copper catalysts exhibited a maximum selectivity toward ethylene (63.1%) and C_2+_ products (80.9%) at −1.01 V versus RHE and showed long‐term durability for over 10 h in eCO_2_RR under neutral conditions. In addition, the catalysts maintained the high FEs and achieved a high partial geometric current density toward C_2+_ products of −255 mA cm^−2^ at −0.62 V versus RHE under alkaline conditions with a gas diffusion electrode system.

We envisioned that designing the precursor morphology and tuning the treatment conditions were particularly effective in generating active catalyst species with optimal performances. Targeting active catalyst species using rational synthetic protocols combined with theoretical and in situ spectroscopic approaches would be able to achieve goals for eCO_2_RR toward economically viable processes with value‐added products.

## 4 Experimental Section

Refer to the Supporting Information for details.

## Conflict of Interest

The authors declare no conflict of interest.

## Author Contributions

S.K. and D.S. contributed equally to this work. S.K. and D.S. designed and carried out all experiments. J.P. measured the ex situ and in situ XAS data. J.‐Y.J. performed the flow cell experiments. S.K., D.S., and H.S. analyzed all data and wrote the manuscript.

## Supporting information

Supporting Information

## Data Availability

The data that support the findings of this study are available from the corresponding author upon reasonable request.
