# Supplementary material for: Grain Boundary‐Rich Copper Nanocatalysts Generated from Metal‐Organic Framework Nanoparticles for CO2‐to‐C2+ Electroconversion
Source: Adv Sci (Weinh). 2023 Jan 22;10(9):2207187. doi: 10.1002/advs.202207187 (PMC10037986; doi:10.1002/advs.202207187)
Supplement: Supplementary file 1 — Supporting Information [file ADVS-10-2207187-s001.pdf]

ADVANCED SCIENCE
Open Access

Supporting Information

for *Adv. Sci.*, DOI 10.1002/advs.202207187

Grain Boundary-Rich Copper Nanocatalysts Generated from Metal-Organic Framework Nanoparticles for CO_2-to-C_{2+} Electroconversion

*Sungjoo Kim, Dongwoo Shin, Jonghyeok Park, Jong-Yeong Jung and Hyunjoon Song**

Supporting Information

Grain Boundary-Rich Copper Nanocatalysts Generated from Metal-Organic Framework Nanoparticles for CO_2-to-C_{2+} Electroconversion

*Sungjoo Kim, Dongwoo Shin, Jonghyeok Park, Jong-Yeong Jung, and Hyunjoon Song**

Methods

Chemicals and Materials: Copper (II) nitrate trihydrate ($Cu(NO_3)_2·3H_2O$, puriss. p.a., 99-104%), trimesic acid (BTC, $C_6H_3(CO_2H)_3$, 95%), 4-methoxybenzoic acid (MeOBA, $CH_3OC_6H_4CO_2H$, 99%), polyvinyl pyrrolidone (PVP, $(C_6H_9NO)_n$, M_w ~55,000), 1,5-pentanediol ($HO(CH_2)_5OH$, 96%), Nafion® perfluorinated resin solution (5_{wt}%, contains 45% water), potassium bicarbonate ($KHCO_3$, >99.95%), lead(II) chloride ($PbCl_2$, 99.999% trace metals basis), potassium chloride solution (~3 M KCl), perchloric acid ($HClO_4$, 70%, 99.999% trace metals basis), deuterium oxide (D_2O, 99.9 atom% D) and dimethyl sulfoxide (DMSO, CH_3SOCH_3, ACS reagent ≥99.9%) were purchased from Sigma Aldrich. 2-Propanol ($(CH_3)_2CHOH$), 99.7%) and methanol (CH_3OH, 99.8%) were purchased from Junsei. Ethanol, acetone and potassium hydroxide (KOH, 93%) were purchased from Daejung. Platinum gauze (100 mesh woven from 0.0762 mm (0.003 in) dia wire, 99.9% (metals basis)) was purchased from Thermo Scientific. Ketjen black carbon powder was purchased from Lion. Carbon paper (Sigracet 39BB) for fabricating gas diffusion electrodes was purchased from Fuel Cell Store. All chemicals were used without further purification.

Synthesis of n- and m-Cu MOFs: For m-Cu MOF, 1.82 g (7.53 mmol) of $Cu(NO_3)_2·3H_2O$ and 0.875 g (4.16 mmol) of BTC were dissolved in 50.0 mL of methanol, respectively. Then, the two precursor solutions were transferred into a 100 mL round-bottom flask and mixed with stirring for 2 h at room temperature.

For n-Cu MOF, 0.966 g (4.00 mmol) of $Cu(NO_3)_2·3H_2O$ was dissolved in 15.0 mL of 1,5-pentanediol in a 30.0 mL vial. 0.560 g (2.67 mmol) of 1,3,5-benzenetricarboxylic acid (BTC) was dissolved in 15.0 mL ethanol in another 30.0 mL vial. 0.183 g (1.20 mmol) of 4-methoxybenzoic acid (MeOBA) was put into a 10.0 mL vial containing 3.00 mL of ethanol and let in a hot water bath (60 °C). 5.30 g of poly(vinylpyrrolidone) (PVP) was dissolved in 15.0 mL of 1,5-pentanediol and preheated at 160 °C in a 100 mL flask. 0.300 mL of the copper precursor solution and 0.300 mL of the BTC solution were injected simultaneously into the flask for seed formation. After 15 min, the remaining copper precursor and BTC solutions were dropwise introduced simultaneously at 1.55 mL min^{-1} using two individual syringes for crystal growth. Immediately, the MeOBA solution was injected into the reaction mixture for termination. After the injection, the temperature was raised to 170 °C and kept for 1.5 h. The flask was then cooled to room temperature.

After the reaction, the reaction mixtures were washed with 200 mL of ethanol and separated by centrifuging at 10,000 rpm for 30 min. The supernatant was decanted, and the washing process was repeated three times. The final precipitates were collected and dispersed in 40.0 mL of ethanol.

Preparation of MDC catalysts: Ketjen black powders were weighed and dispersed in ethanol (5.00 mg mL^{-1}) by sonication for 1 h. Then, the Cu-MOF dispersion was introduced into the mixture, targeting 30 wt % Cu to the entire catalyst mass. After 1 h stirring, the catalyst samples were separated by centrifugation at 10,000 rpm for 20 min and washed with 45.0 mL of acetone. The catalysts were dried in a vacuum oven at 45 °C overnight. The catalyst powders were ground before calcination. n-Cu MOF samples were calcined at 225 °C, 250 °C, and 300 °C for 3 h (n-MDC-225, 250, and 300). m-Cu MOF samples were calcined at 250 °C, 265 °C, and 300 °C (m-MDC-250, 265, and 300) for 3 h.

Characterization: Transmission electron microscopy (TEM) and high-resolution transmission electron microscopy (HRTEM) images were obtained using FEI Tecnai G2 F30 S-Twin. The HRTEM images and corresponding fast Fourier transformation (FFT) patterns were processed with Gatan GM3 software for inverse FFT mapping images. Scanning transmission electron microscopy-annular dark field (STEM-ADF) imaging and energy-dispersive X-ray spectroscopy (EDS) elemental mapping analysis were carried out using FEI Talos F200X. Scanning transmission electron microscopy-electron energy loss spectroscopy (STEM-EELS) was carried out using FEI Titan cubed G2 60-300. The mapping images of copper oxidation states using STEM-EELS were abstracted by simultaneous standard quantification with Gatan GM3 software. Scanning electron microscopy (SEM) images were acquired from Hitachi SU5000. X-ray diffraction (XRD) was measured by Rigaku SmartLab, and the spectra processing and crystalline size estimation were performed using SmartLab Studio II software. The inductively coupled plasma-optical emission spectroscopy (ICP-OES, 5110 ICP-OES, Agilent) was used to measure the Cu content. A thermogravimetric analyzer (TGA Q50, TA Instruments) was utilized to investigate the thermal behaviors of the samples. The analysis was conducted under a 60 mL min^{-1} N_2 balance flow and a 40 mL min^{-1} air sample flow with a temperature increment rate of 5 °C min^{-1}. X-ray photoelectron spectroscopy (XPS) was conducted on Thermo VG Scientific K-alpha.

eCO_2RR Study in the H-cell under the neutral condition: For fabricating the working electrode, the catalyst was dispersed in 2-propanol to fix the concentration of 1.00 mg_{cat} mL^{-1} and sonicated for 1 h to yield a catalyst ink dispersion. A 120 μL of the ink dispersion was drop cast onto a polished glassy carbon electrode (d = 7.98 mm, catalyst mass loading of 240 mg cm^{-2}). Then, 70.0 μL of a diluted Nafion solution (50.0 μL of Nafion perfluorinated resin solution in 0.950 mL of 2-propanol) was loaded onto the electrode to immobilize the catalyst.

All electrochemical performances were measured using a CHI 760E potentiostat workstation (CH Instruments, Inc.) with a three-electrode system. A handmade H-cell was used for electrochemical operation, where the catholyte and anolyte were separated and contacted through a proton permeable membrane (Nafion 117, Sigma Aldrich). The volume of each part was 220 mL, filled with 80 mL of a 0.1 M $KHCO_3$ electrolyte. Ag/AgCl (3 M NaCl) (+0.209 V vs. standard hydrogen electrode) and Pt plate electrodes were used as reference and counter electrodes. All electrochemical measurements were performed in 0.1 M $KHCO_3$ aqueous solution, purged with CO_2 gas for 1 h before the experiments. The electrolyte pH was fixed to be 6.87. The catalyst electrode was activated for 10 min at −1.35 V vs. RHE to stabilize chronoamperometry signals. A scan rate of 0.1 V s^{-1} was applied for linear sweep voltammetry (LSV). Electrochemical impedance spectroscopy (EIS) data were obtained at the open-circuit voltage in a frequency range of 10,000 kHz to 0.1 Hz. The currents of eCO_2RR were measured by chronoamperometry at each potential. All electrochemical data were obtained with CO_2 gas bubbling at 60 mL min^{-1}. The potential values were corrected to RHE using the equation ($E_{RHE} = E_{Ag/AgCl} + 0.209 + 0.059 \times pH$). All electrochemical results were *iR*-corrected.

eCO_2RR study in the flow cell under the alkaline condition: The flow cell electrodes were prepared by air-brushing the catalysts on carbon paper with a 4.0 cm^2 geometric area. The catalyst samples were dispersed in fresh ethanol in separate vials with 15 min sonication to obtain 1.0 mg mL^{-1} catalyst ink. Then, the ink was sprayed onto the carbon paper to target a loading amount of 500 μg cm^{-2}. Nafion solution (manufactured by dissolving 30 μL Nafion perfluorinated resin solution into 3.0 mL of 2-propanol) was then air-brushed onto the GDL and allowed to dry at room temperature.

A handmade GDE flow cell was utilized to measure the electrocatalytic performances of MDC samples. All the electrochemical data were obtained by using BioLogic SP-150 potentiostat. A typical three-electrode system was constructed with a

Ag/AgCl (3 м NaCl) reference electrode and Pt gauze counter electrode. The GDE hindered by a 1.0 cm^2 geometrical area was set to be the working electrode. The catholyte and anolyte compartments were separated by Nafion 117 (Sigma Aldrich) cation exchange membrane. The CO_2 flow rate was set to be 15 sccm, and 100 mL of 1.0 м KOH catholyte and anolyte contained in separate chambers were kept flowing at a 15 mL min^{-1} rate during the operation. Before the product analysis, the catalyst-loaded GDE was electrochemically activated for 30 min at −0.74 V vs. RHE. Chronopotentiometry mode was set to fix the total current density applied onto the system for 30 min. Potentiostatic electrochemical impedance spectroscopy (PEIS) was inserted between the steps to determine ohmic resistances. The initial and final pHs of the catholyte were measured with a pH meter, and pH after each step was estimated by linear scaling. All electrochemical potentials were entirely *iR* compensated and corrected to RHE.

ECSA measurements: The electrochemically active surface areas (ECSAs) of the catalyst samples were determined by Pb stripping. The electrolyte for Pb underpotential deposition and stripping experiments was prepared by dissolving 0.0278 g (0.100 mmol) of $PbCl_2$ into the mixture of $HClO_4$ (1.73 mL), 3 M KCl solution (3.30 mL) and deionized water (195 mL). The electrolyte was purged and saturated with argon flow for 2 h before the electrochemical measurement. The three-electrode system and electrolyte volume were set to be above conditions in the H-cell. Cyclic voltammetry was repeatedly conducted between 0.00 V and −0.50 V vs. Ag/AgCl (3 м NaCl) electrode with a scan rate of 0.01 V s^{-1}.

XAS measurements: X-ray absorption spectroscopy (XAS) data were collected at Pohang Accelerator Laboratory (7D-XAFS beamline in PLS-II). The beamline was set to have an energy of 2.5 GeV and a ring current of 300 mA using Si(111) as monochromators. The measurement ranges for Cu K-edge XAS were set from 8879 eV to 9837 eV. The X-ray absorption near edge structure (XANES) region was acquired with a step of 0.4 eV and 2.0 s duration, and extended X-ray absorption fine structure (EXAFS) was obtained with a step size of 0.3 nm^{-1} for 3.0 s. The linear combination fitting of XANES was conducted by Athena software. Artemis software included in the Demeter package for EXAFS fitting analyses was used after data processing was performed with Athena. The background removal was done by AUTOBK program with R_{bkg} = 0.10 nm. The k^2- or k^3-weighting and Kaiser-Bessel window functions were chosen for fitting the Fourier-transformed EXAFS data

in k space. Single scattering paths from the model crystalline structure were extracted from the FEFF calculation. The amplitude reduction factor (S_0^2) for Cu was 0.94, obtained from the fitting result of Cu foil EXAFS data under the identical condition of sample measurement. The goodness of fitting was determined from the R-factor available from the refinement.

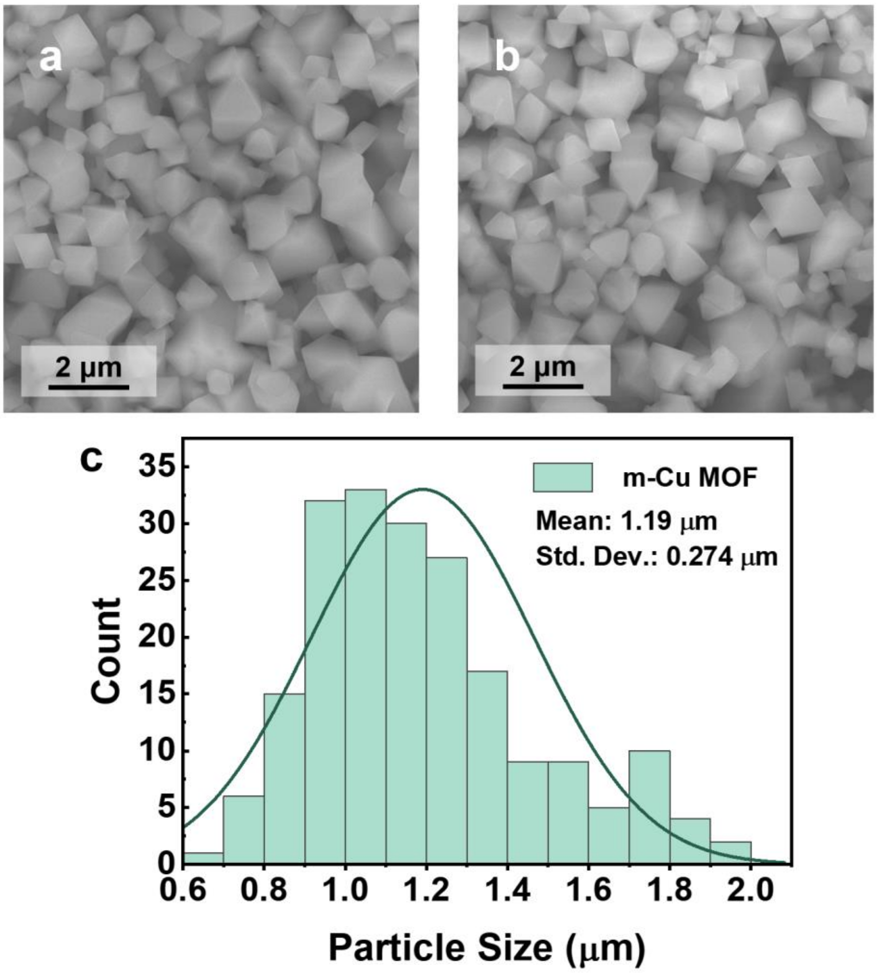

Figure S1. a-b) SEM images of m-Cu MOF. The scale bars represent 2 µm. c) A particle size distribution histogram of m-Cu MOF. The cyan line is a Gaussian fit with an average diameter of 1.19 µm and a standard deviation of 0.274 µm.

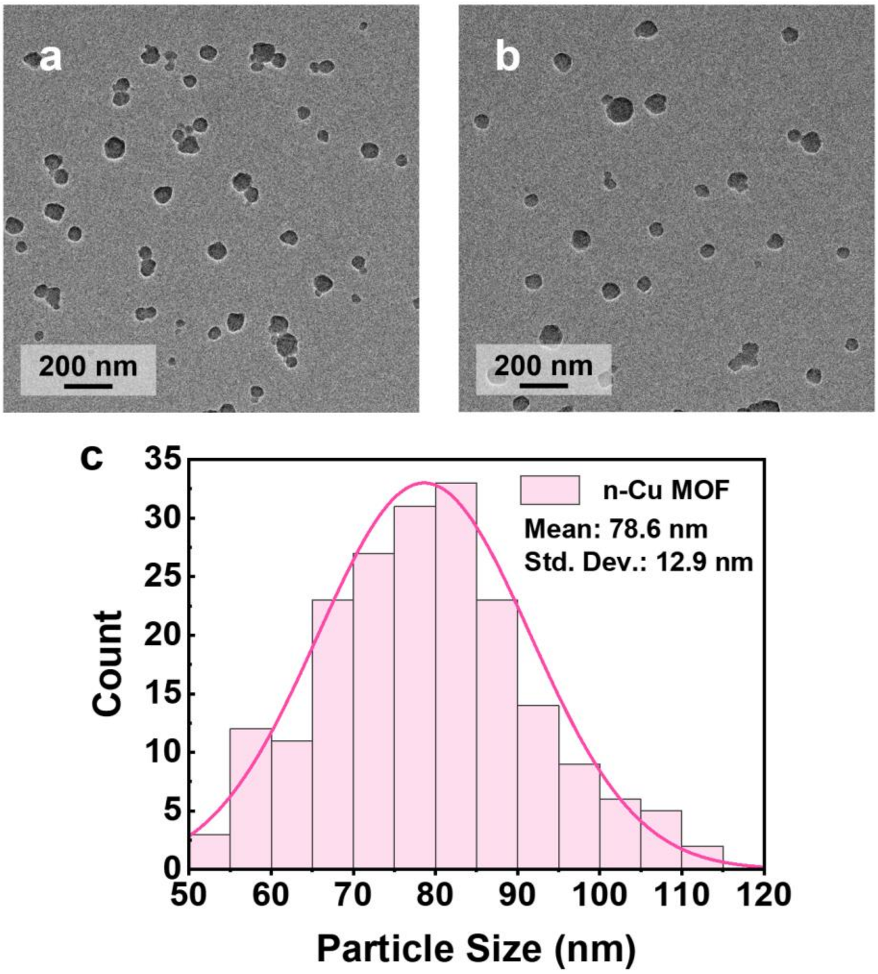

Figure S2. a-b) TEM images of n-Cu MOF. The scale bars represent 200 nm. c) A particle size distribution histogram of n-Cu MOF. The red line is a Gaussian fit with an average diameter of 78.6 nm and a standard deviation of 12.9 nm.

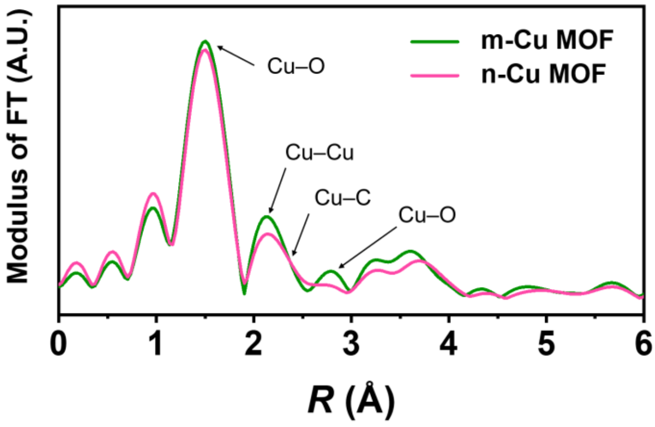

Figure S3. Fourier-transformed magnitudes of Cu K-edge EXAFS spectra for m- and n-Cu MOF. The structural parameters of the best fits are summarized in Table S1.

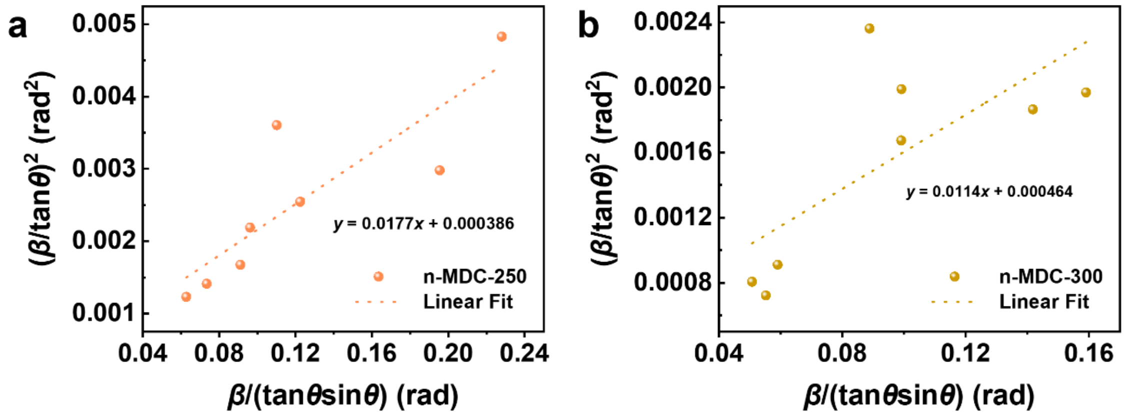

Figure S4. Halder-Wagner plots for a) n-MDC-250 and b) n-MDC-300. Seven XRD peaks corresponding to (110), (002), (20$\bar{2}$), (020), (202), (11$\bar{3}$), (31$\bar{1}$), and (220) were selected for each plot. The θ and β indicate the Bragg angle and the full width at half maximum (FWHM) of each peak, respectively. Detailed estimated crystalline size and strain values are summarized in Table S3.

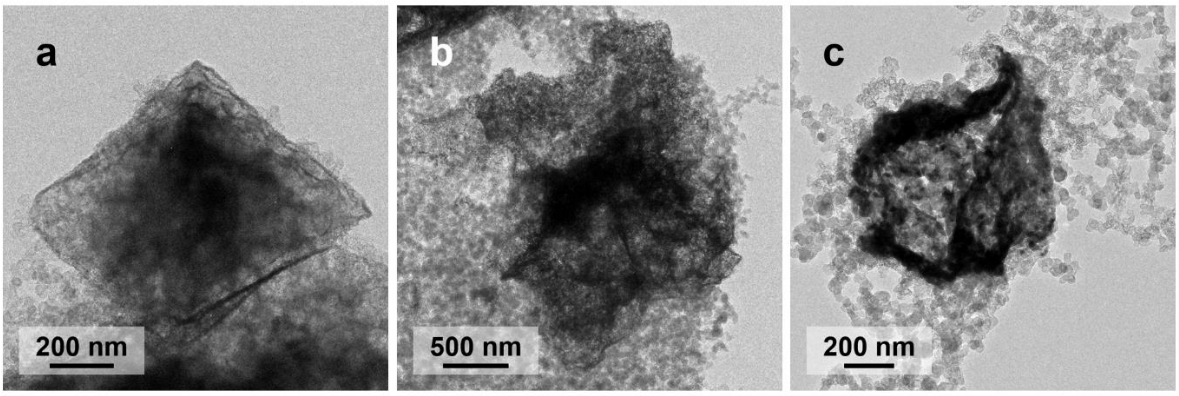

Figure S5. TEM images of a) m-MDC-250, b) m-MDC-265, and c) m-MDC-300.

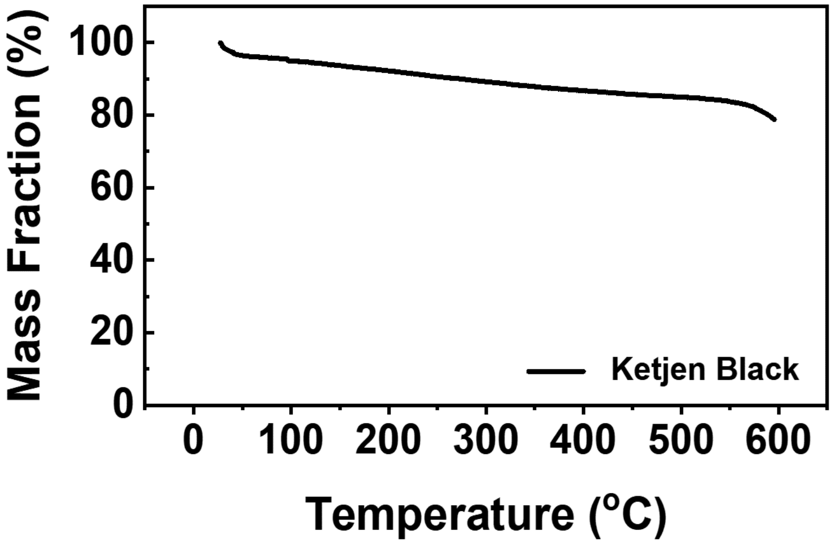

Figure S6. A TGA profile of bare Ketjen black.

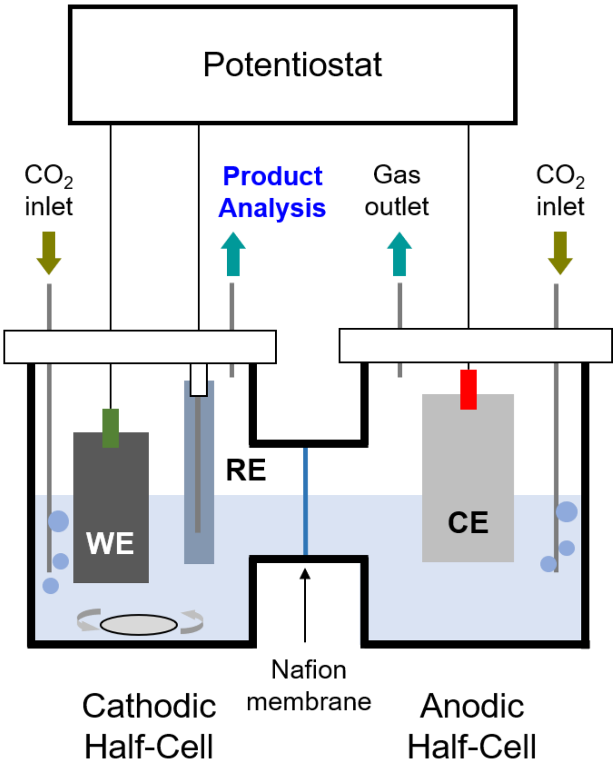

Figure S7. A schematic representation of a handmade H-cell system used for eCO_2RR study.

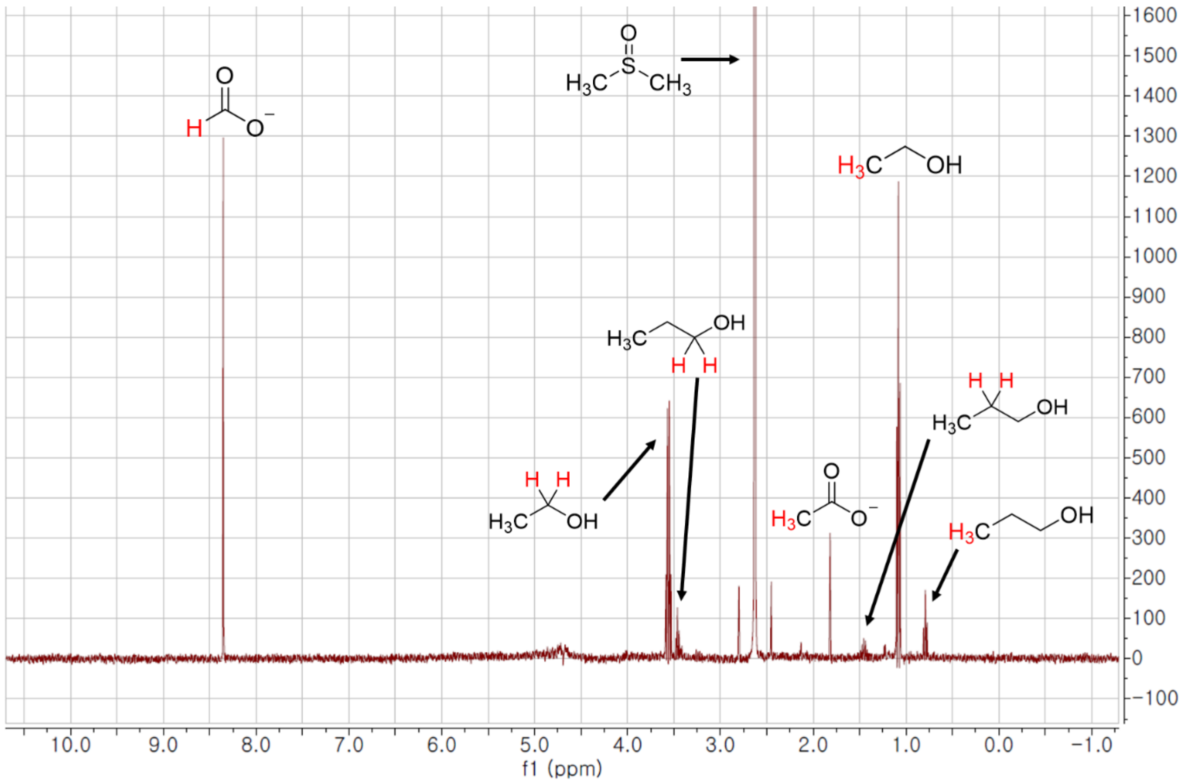

Figure S8. Representative ^{1}H-NMR spectrum for quantifying liquid products generated from eCO_2RR. Dimethyl sulfoxide (DMSO) was used as an internal standard. The proton peaks assigned to corresponding molecular structures are present on the spectrum as red marks.

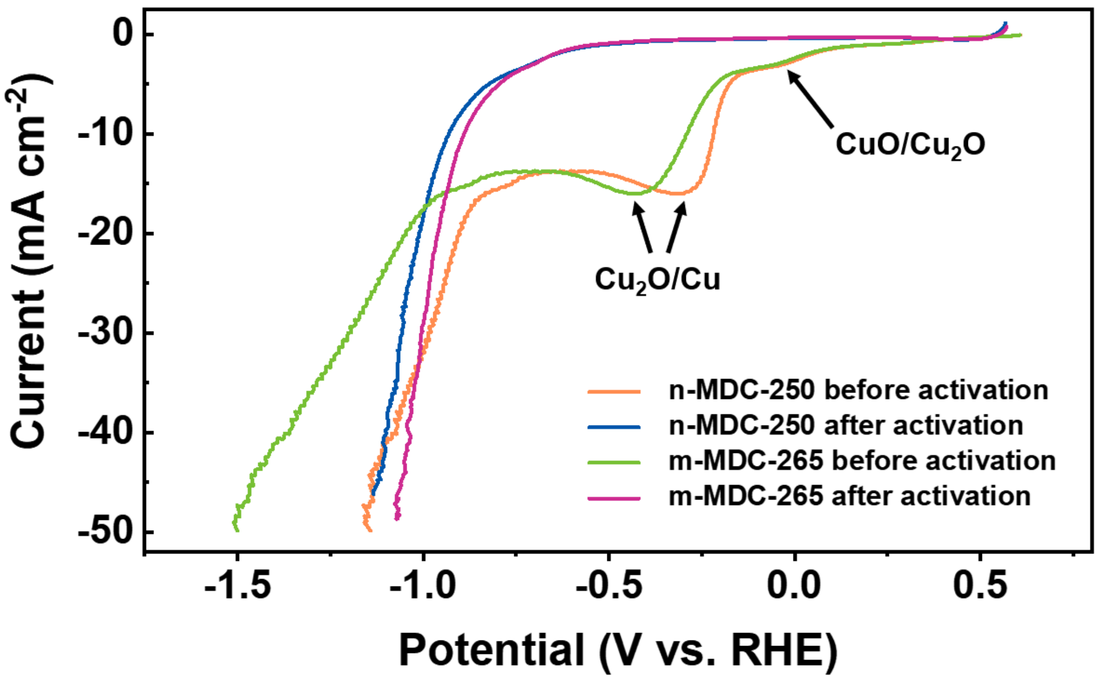

Figure S9. LSV curves of n-MDC-250 and m-MDC-265 before and after the electrochemical activation. The reduction peaks at −0.02 V vs. RHE correspond to a reduction of CuO to Cu_2O. The peaks at −0.3–0.4 V vs. RHE match the reduction of Cu_2O to Cu.[1-2]

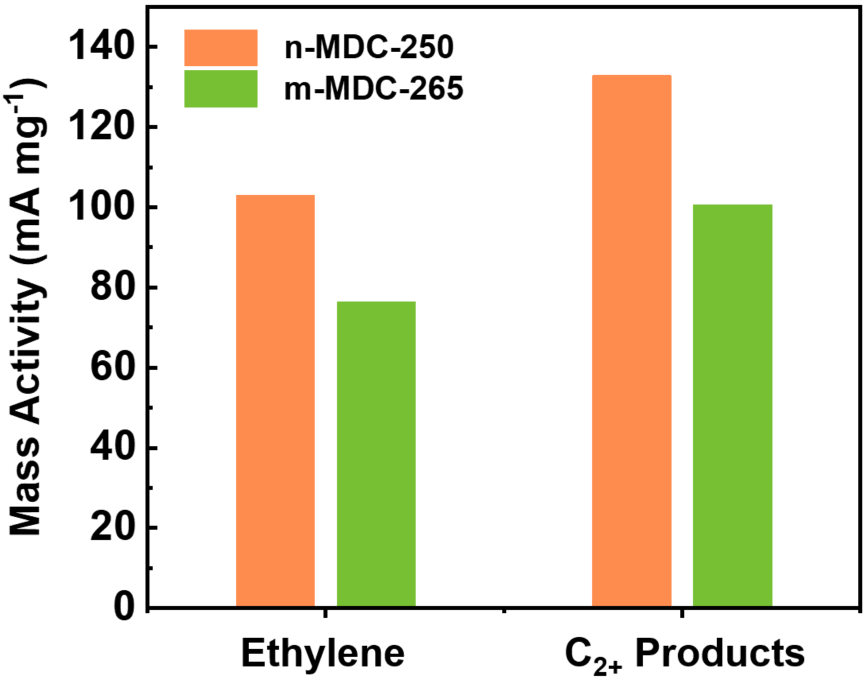

Figure S10. Mass activities of n-MDC-250 and m-MDC-265 toward ethylene and C_{2+} products in eCO_2RR.

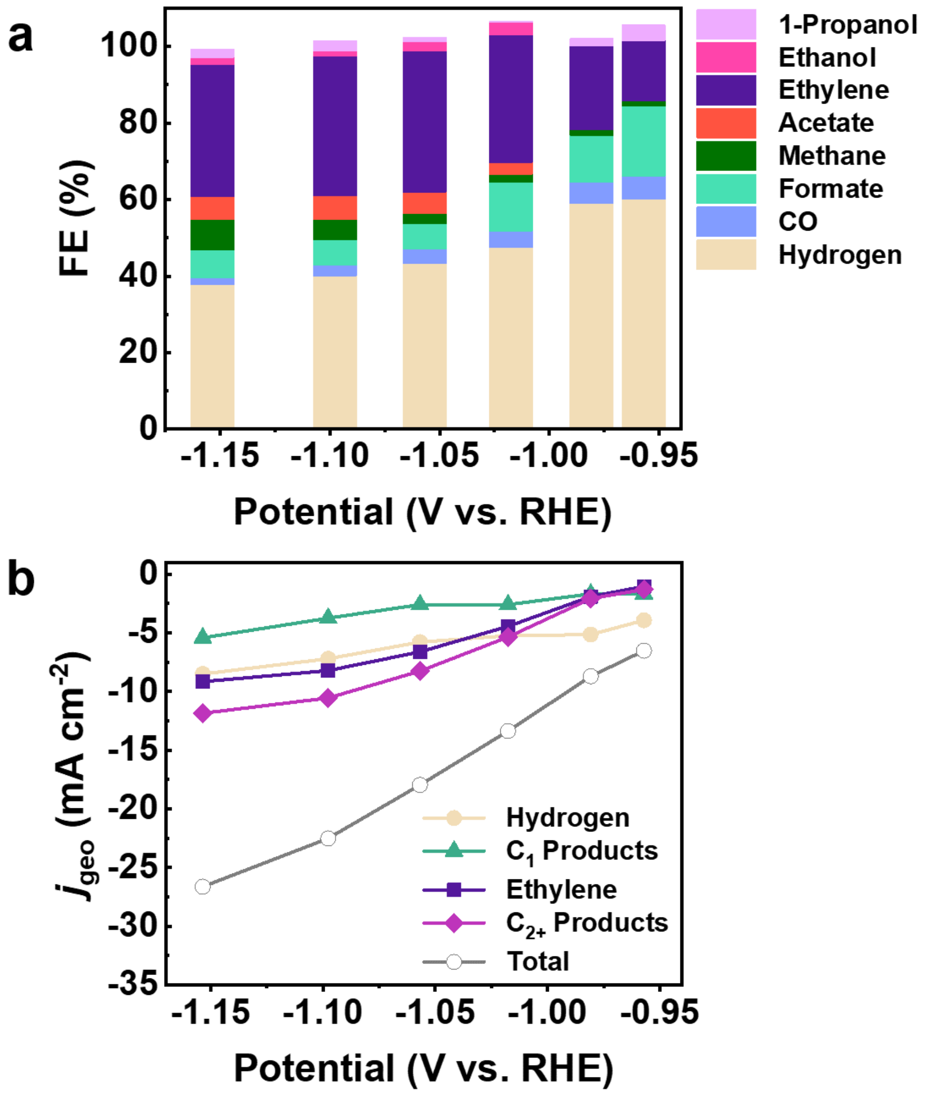

Figure S11. a) FEs and b) geometric current densities of products vs. applied potentials using commercial CuO nanoparticles (80 nm in diameter).

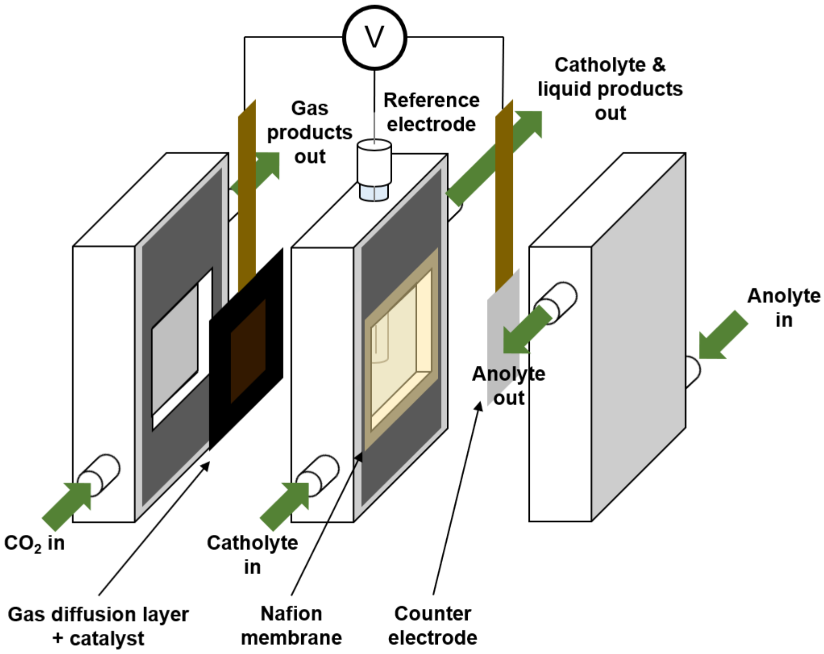

Figure S12. A schematic representation of a handmade GDE flow cell system used for eCO_2RR study.

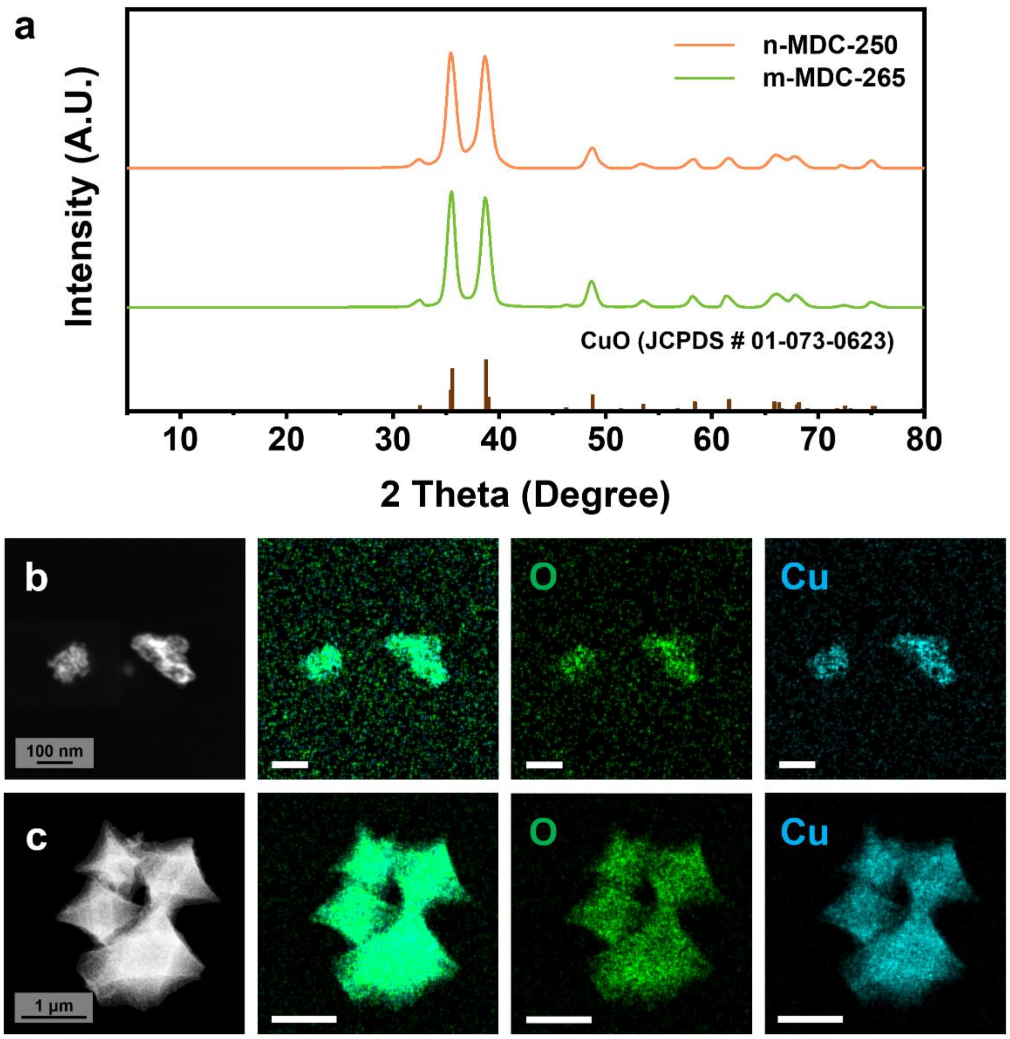

Figure S13. a) Powder XRD patterns of n-MDC-250 and m-MDC-265 without Ketjen black carbon support. STEM-HAADF and STEM-EDS elemental mapping images of b) n-MDC-250 (scale bars = 100 nm) and c) m-MDC-265 (scale bars = 1 μm) without carbon supports. The O and Cu elements are colored in green and blue.

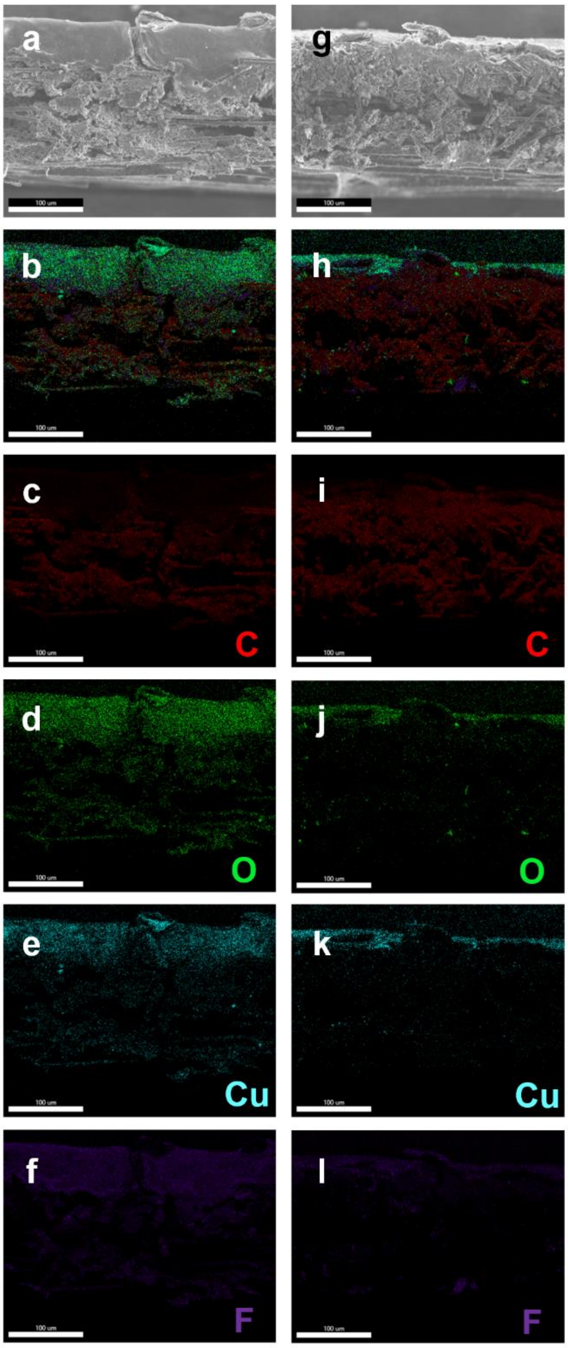

Figure S14. a) Cross-sectional SEM and b-f) EDS elemental mapping images of n-MDC-250 loaded on the carbon paper. g) Cross-sectional SEM and h-i) EDS elemental mapping images of m-MDC-265 loaded on the carbon paper. The C, O, Cu, and F elements are colored red, green, blue, and violet. F originates from the Nafion ionomer. The scale bars represent 100 μm.

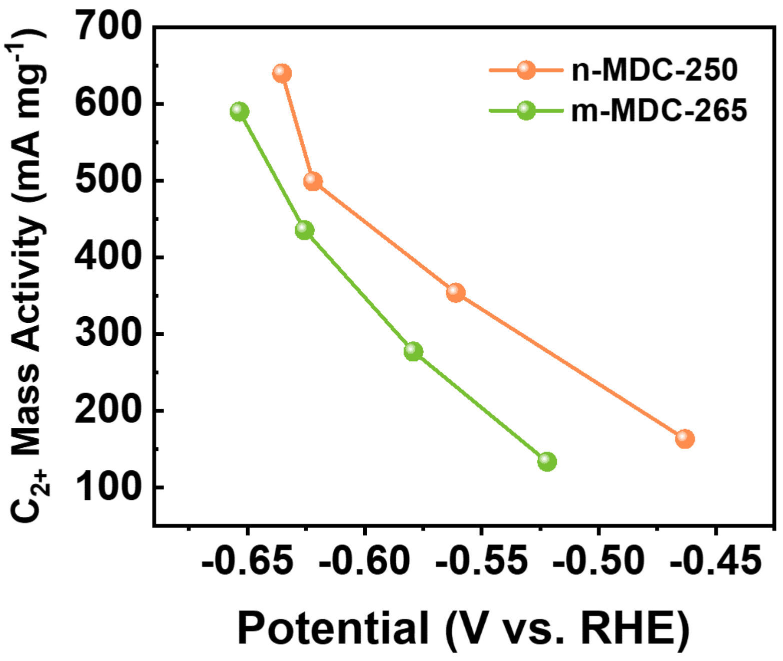

Figure S15. Mass activities of n-MDC-250 and m-MDC-265 against various applied potentials acquired from the gas diffusion flow cell configuration.

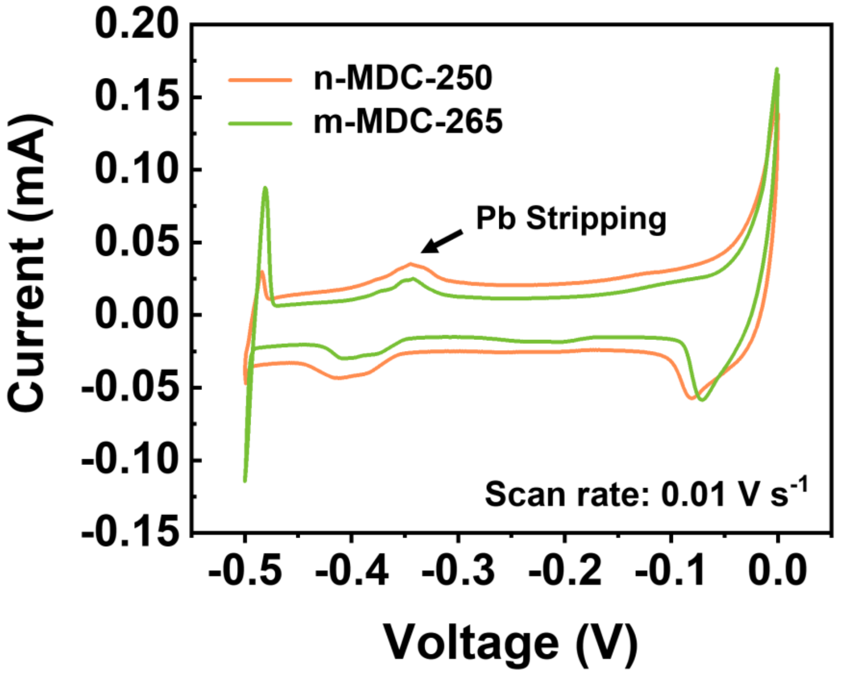

Figure S16. Cyclic voltammograms of Pb underpotential deposition and stripping of n-MDC-250 and m-MDC-265 with the scan rates of 0.01 V s^{-1}.

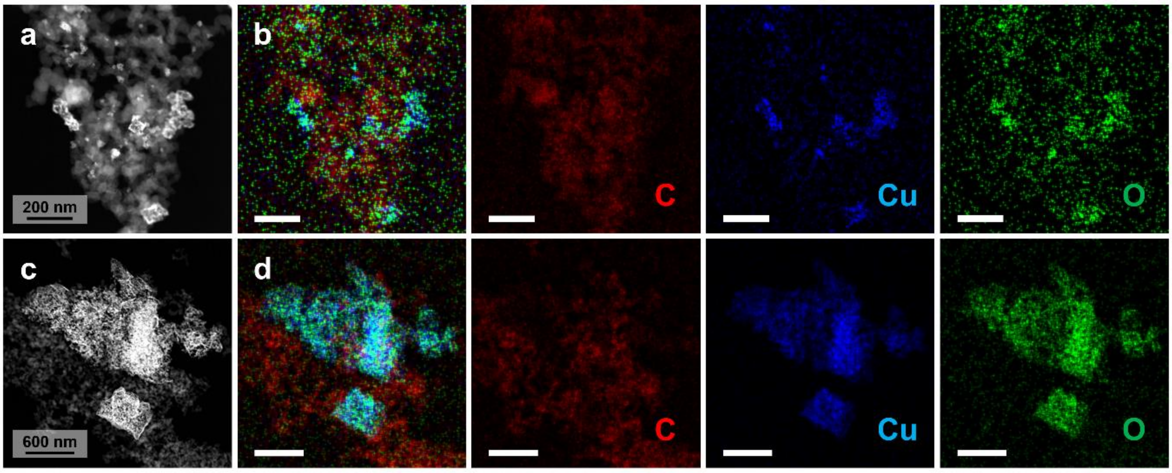

Figure S17. a) STEM-HAADF and b) STEM-EDS elemental mapping images of n-MDC-250 before the activation. c) STEM-HAADF and d) STEM-EDS elemental mapping images of m-MDC-265 before the activation. C, Cu, and O were mapped by red, blue, and green colors.

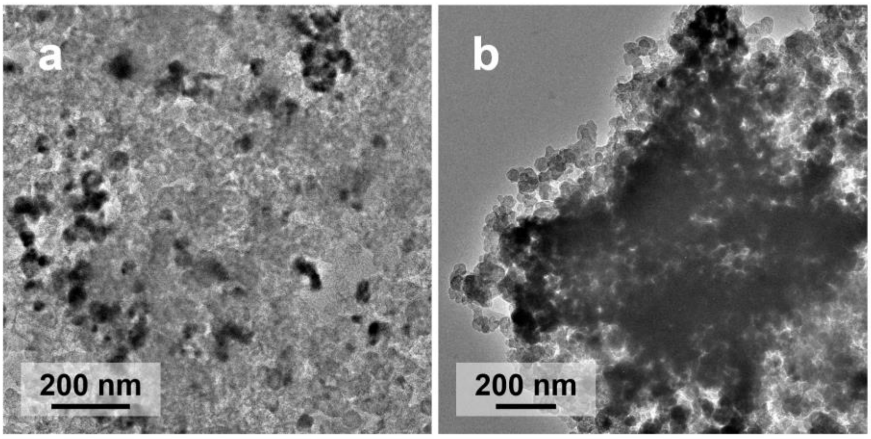

Figure S18. TEM images of a) n-MDC-250 and b) m-MDC-265 after the 12 h operation of eCO_2RR.

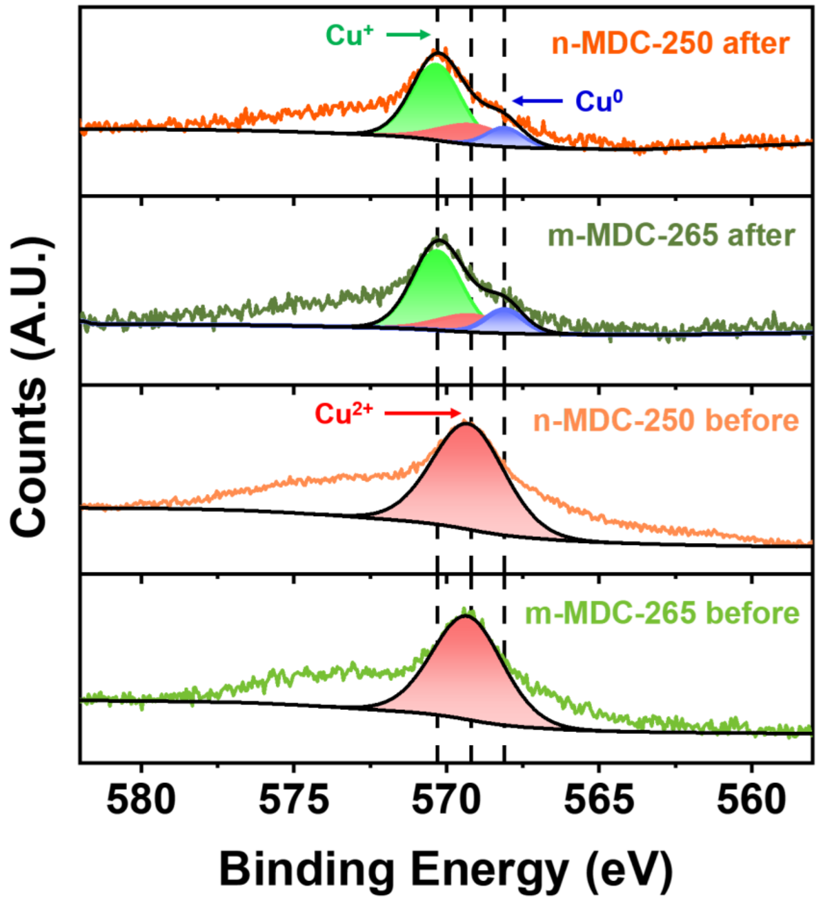

Figure S19. Cu LMM Auger electron spectra of n-MDC-250 and m-MDC-265 before and after the activation.

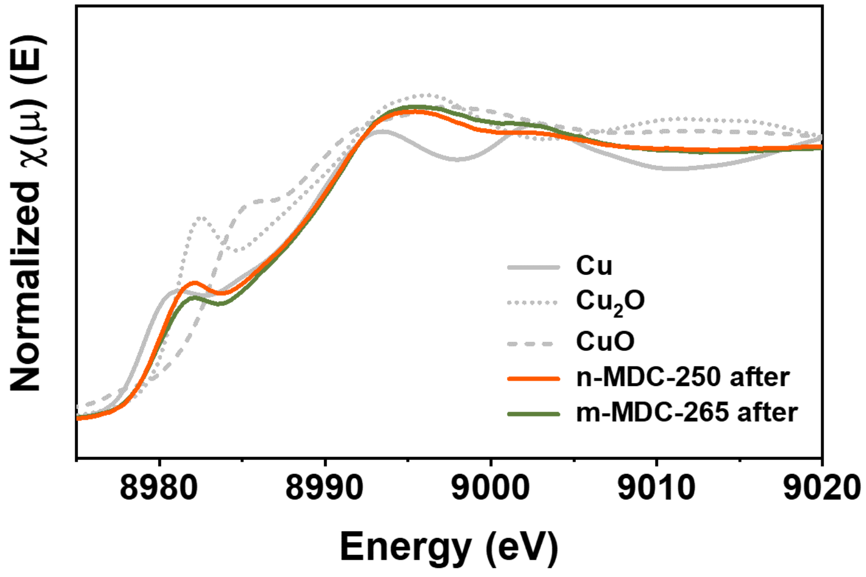

Figure S20. ex-situ Cu K-edge XANES spectra of n-MDC-250 and m-MDC-265 after the activation. The linear combination fitting results of XANES spectra are summarized at Table S14.

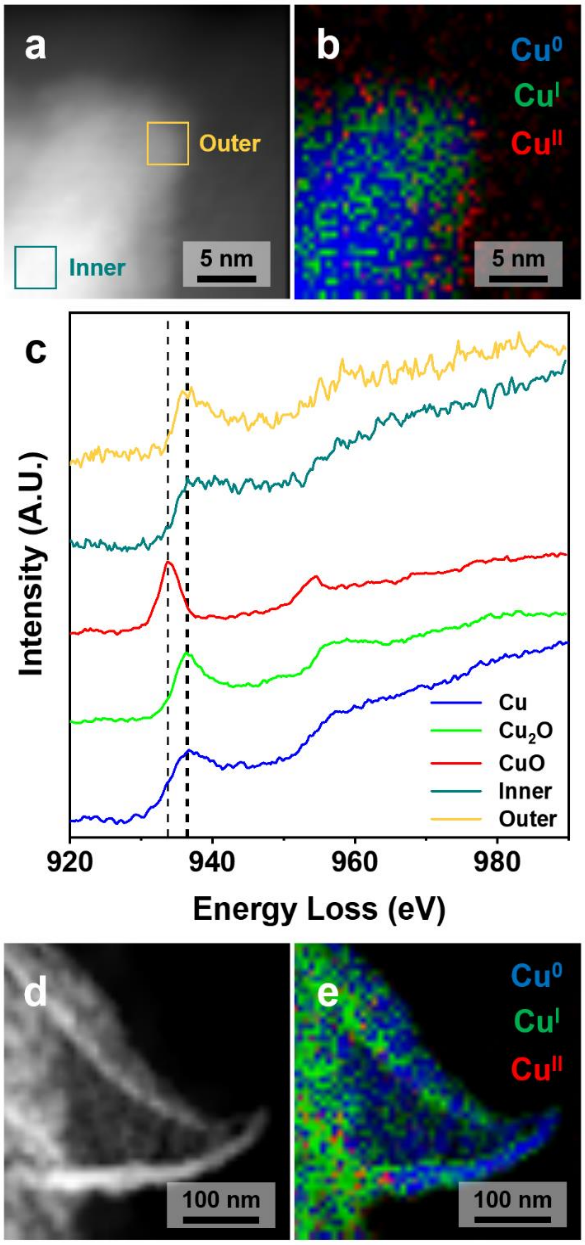

Figure S21. a) STEM-ADF and b) corresponding STEM-EELS Cu oxidation state mapping images of n-MDC-250 after the activation. c) Energy loss near edge structure (ELNES) profiles of the inner and outer parts of the n-MDC-250 sample with references. d) STEM-ADF and e) corresponding STEM-EELS Cu oxidation state mapping images of m-MDC-265 after the activation. The oxidation state mapping images were obtained by the concurrent standard quantification method.[3-4] The ELNES peaks appeared at 936.9 eV, 936.3 eV, and 933.9 eV for Cu metal, Cu_2O, and CuO, respectively.

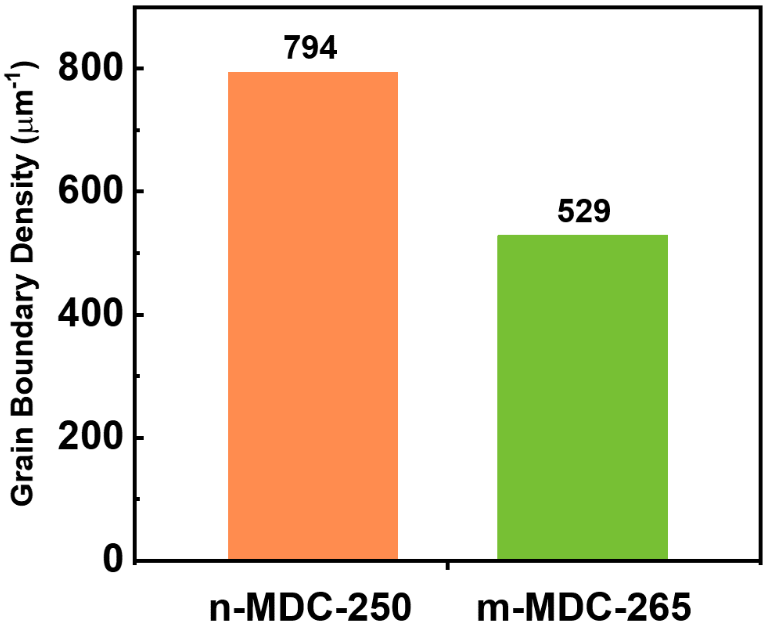

Figure S22. Grain boundary densities of n-MDC-250 and m-MDC-265 estimated by the inverse FFT mapping analysis.

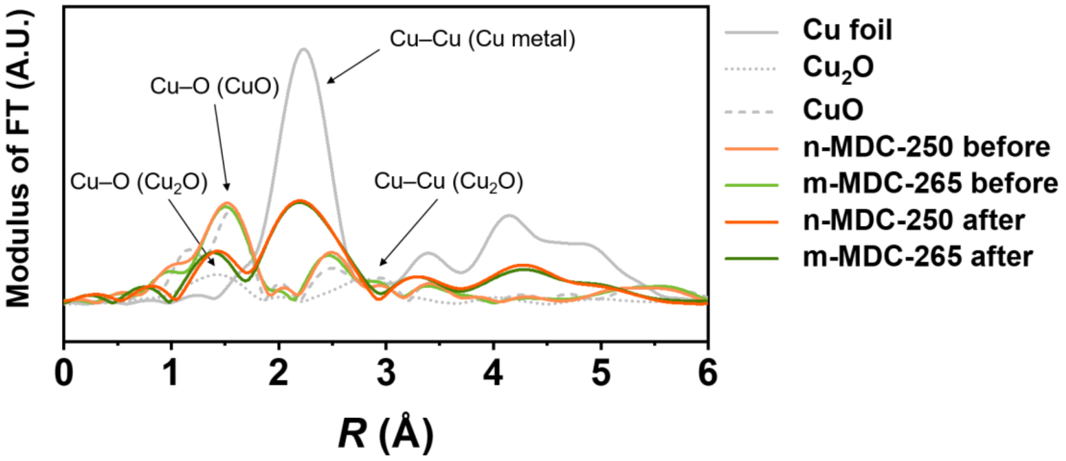

Figure S23. Fourier-transformed magnitudes of Cu K-edge EXAFS spectra for n-MDC-250 and m-MDC-265 before and after activation with Cu, Cu_2O, and CuO references. The structural parameters of the best fits are summarized in Table S15 and S16.

Table S1. Summary of structural parameters of m- and n-Cu MOF samples acquired from Cu K-edge EXAFS

Sample	Shell	CN[a)]	R [nm][b)]	σ^2 [nm^2][c)]	R-factor
m-Cu MOF	Cu–O_1	4.00	0.193 ± 0.000	46.7 ± 3.4	0.00925
	Cu–Cu_1	1.00	0.241 ± 0.008	71.2 ± 7.2	
	Cu⋯C_1	4.00	0.260 ± 0.013	10.4	
	Cu– C–O_1	4.00	0.292 ± 0.006	174.6	
	Cu–O_2	8.00	0.313 ± 0.003	105.5 ± 24.7	
n-Cu MOF	Cu–O_1	4.00	0.195 ± 0.002	48.1 ± 6.2	0.0171
	Cu–Cu_1	1.00	0.248 ± 0.001	127.5 ± 99.8	
	Cu⋯C_1	4.00	0.266 ± 0.013	60.5	
	Cu– C–O_1	4.00	0.294 ± 0.006	85.3	
	Cu–O_2	8.00	0.302 ± 0.003	227.3 ± 125.0	

[a)] CN refers to the coordination number; [b)] R represents the interatomic distance; [c)] σ^2 describes the Debye Waller factor. The fitting ranges were set to be $1.15 \leq R$ (Å) ≤ 3.10. The single scattering pathways and obtuse triangular pathways were obtained from the FEFF calculation of HKUST-1 crystalline structure.

Table S2. Crystalline domain size estimation of n-MDC-250 and n-MDC-300 from the Scherrer's equation

Plane	Sample	Bragg Angle [°]	FWHM [°]	Average Domain Size [nm]
(110)	n-MDC-250	32.42	0.8535	9.69
	n-MDC-300	32.41	0.5761	14.4
(002)	n-MDC-250	35.49	0.9445	8.83
	n-MDC-300	35.46	0.5697	14.6
Mean	n-MDC-250	.	.	9.26
	n-MDC-300	.	.	14.5

The X-ray source for the analyses was Cu Kα_1, 1.54 Å.

Table S3. Crystalline domain size estimation of n-MDC-250 and n-MDC-300 from the Halder-Wagner's method

Sample	Strain [%]	Average Domain Size [nm]
n-MDC-250	0.491	9.14
n-MDC-300	0.539	14.2

The X-ray source for the analyses was Cu Kα_1, 1.54 Å.

Table S4. The Cu weight percentage of the catalyst samples measured by ICP-OES

Sample	Calcination Temperature [°C]	Weight percentage of Cu [%]
n-MDC	225	33.6
	250	29.3
	300	37.0
m-MDC	250	29.7
	265	26.8
	300	28.4

Table S5. FEs and standard deviations of n-MDC-250 measured at various applied potentials in 0.1 M $KHCO_3$

Potential [V vs. RHE]	FE [%]								
	Hydrogen	Formate	CO	Methane	Acetate	Ethylene	Ethanol	1-Propanol	Total
−0.97	14.8 ± 4.4	8.0 ± 1.0	4.2 ± 1.1	1.3 ± 0.3	1.0 ± 1.3	59.3 ± 3.1	8.6 ± 1.2	5.1 ± 1.4	102.3 ± 2.6
−1.01	13.4 ± 6.5	3.2 ± 0.3	2.3 ± 0.8	1.9 ± 0.6	1.2 ± 0.8	63.1 ± 1.9	12.5 ± 3.0	4.1 ± 1.2	101.7 ± 4.0
−1.05	23.8 ± 3.1	2.4 ± 0.6	1.6 ± 0.4	4.1 ± 3.1	0.6 ± 0.6	57.8 ± 2.5	8.8 ± 1.7	3.0 ± 1.4	102.1 ± 1.5
−1.09	34.3 ± 2.7	1.2 ± 0.1	1.2 ± 0.6	6.7 ± 4.2	1.0 ± 0.5	45.9 ± 7.6	7.6 ± 1.8	1.2 ± 0.6	99.1 ± 5.2
−1.13	53.6 ± 4.5	1.2 ± 0.3	0.7 ± 0.2	8.8 ± 3.4	0.8 ± 0.5	30.2 ± 2.0	6.8 ± 0.7	0.4 ± 0.4	102.4 ± 5.1
−1.16	61.6 ± 4.5	0.4 ± 0.1	0.6 ± 0.2	9.6 ± 3.2	0.8 ± 0.3	21.2 ± 0.8	5.0 ± 0.9	1.1 ± 1.3	100.2 ± 1.1

Table S6. FEs of n-MDC families in 0.1 M $KHCO_3$ condition at −1.01 V vs. RHE

Catalyst	FE [%]								
	Hydrogen	Formate	CO	Methane	Acetate	Ethylene	Ethanol	1-Propanol	Total
n-MDC-225	45.9	5.8	7.3	9.8	0.0	29.6	5.2	0	103.6
n-MDC-250	13.4	3.2	2.3	1.9	1.2	63.1	12.5	4.1	101.7
n-MDC-300	22.2	9.6	5.0	0.5	7.7	52.6	2.0	1.9	101.5

Table S7. FEs and standard deviations of m-MDC-265 measured at various applied potentials in 0.1 м $KHCO_3$

Potential [V vs. RHE]	FE [%]								
	Hydrogen	Formate	CO	Methane	Acetate	Ethylene	Ethanol	1-Propanol	Total
−0.97	29.6 ± 0.4	8.1 ± 1.2	4.2 ± 2.6	0.9 ± 0.4	0.9 ± 0.7	44.4 ± 2.1	9.1 ± 1.1	3.2 ± 2.2	100.4 ± 0.4
−1.01	27.7 ± 3.8	5.9 ± 2.3	2.7 ± 1.6	1.4 ± 0.6	0.1 ± 0.1	48.3 ± 1.9	11.6 ± 1.5	2.9 ± 1.9	100.5 ± 1.0
−1.04	31.2 ± 5.0	3.9 ± 1.2	2.0 ± 1.2	1.7 ± 0.7	0.6 ± 0.2	49.1 ± 4.0	9.2 ± 3.6	3.5 ± 3.6	101.2 ± 2.1
−1.09	41.8 ± 6.7	1.5 ± 0.4	1.4 ± 0.9	3.1 ± 1.1	0.4 ± 0.4	41.7 ± 4.2	9.8 ± 1.4	1.3 ± 1.4	100.9 ± 1.3
−1.14	52.8 ± 4.0	1.4 ± 0.7	1.0 ± 0.6	6.4 ± 2.4	0.4 ± 0.4	29.8 ± 2.0	8.3 ± 1.0	2.3 ± 1.0	102.4 ± 2.3
−1.19	60.1 ± 4.5	1.1 ± 0.4	0.9 ± 0.6	9.0 ± 3.6	0.6 ± 0.5	21.6 ± 3.5	6.1 ± 1.3	0.4 ± 1.3	99.6 ± 3.8

Table S8. FEs of m-MDC families in 0.1 м $KHCO_3$ condition at −1.01 V vs. RHE

Catalyst	FE [%]								
	Hydrogen	Formate	CO	Methane	Acetate	Ethylene	Ethanol	1-Propanol	Total
m-MDC-250	36.0	11.7	10.4	3.3	0.0	34.7	5.6	0.0	101.7
m-MDC-265	27.7	5.9	2.7	1.4	0.1	48.3	11.6	2.9	100.5
m-MDC-300	27.8	24.6	7.7	0.7	6.0	25.1	7.9	3.1	102.9

Table S9. FEs and standard deviations of n-MDC-250 tested for various applied j_{tot} in the GDE flow cell with 1.0 м KOH electrolyte condition

j_{tot} [mA cm^{-2}]	FE [%]								
	Hydrogen	Formate	CO	Methane	Acetate	Ethylene	Ethanol	1-Propanol	Total
−400	19.3 ± 1.8	1.7 ± 0.2	9.7 ± 0.8	3.4 ± 1.1	5.9 ± 0.5	41.6 ± 2.2	13.8 ± 1.8	2.5 ± 1.1	98.0 ± 2.2
−300	17.9 ± 0.8	2.3 ± 0.5	8.7 ± 1.1	3.5 ± 0.7	6.6 ± 1.9	43.2 ± 1.7	14.9 ± 0.2	1.7 ± 0.2	98.9 ± 0.3
−200	15.2 ± 3.1	3.0 ± 0.9	9.1 ± 0.9	3.6 ± 0.4	8.2 ± 2.1	45.6 ± 1.6	13.9 ± 3.3	3.0 ± 0.9	101.6 ± 1.5
−100	19.8 ± 0.2	4.2 ± 0.3	10.7 ± 2.5	2.9 ± 0.8	12.6 ± 5.5	40.2 ± 0.6	7.5 ± 3.3	4.9 ± 0.9	102.8 ± 1.5

Table S10. FEs and standard deviations of m-MDC-265 tested for various applied j_{tot} in the GDE flow cell with 1.0 м KOH electrolyte condition

j_{tot} [mA cm^{-2}]	FE [%]								
	Hydrogen	Formate	CO	Methane	Acetate	Ethylene	Ethanol	1-Propanol	Total
−400	24.4 ± 2.8	2.5 ± 0.9	8.4 ± 1.5	5.6 ± 1.3	7.0 ± 2.4	38.6 ± 4.5	11.0 ± 1.8	2.4 ± 0.9	99.8 ± 3.4
−300	27.0 ± 1.8	2.6 ± 1.0	8.2 ± 0.2	5.9 ± 0.2	7.5 ± 3.2	35.9 ± 2.1	11.5 ± 0.2	3.1 ± 1.0	101.6 ± 1.8
−200	25.6 ± 5.9	4.1 ± 1.5	11.1 ± 2.5	5.7 ± 0.7	7.7 ± 3.9	37.7 ± 5.6	7.4 ± 1.6	2.6 ± 1.9	101.9 ± 2.5
−100	26.3 ± 3.0	6.1 ± 2.3	13.6 ± 3.1	4.5 ± 1.7	9.2 ± 1.5	33.1 ± 6.9	7.1 ± 2.6	4.0 ± 0.7	104.0 ± 0.7

Table S11. Comparison of copper catalysts derived from Cu-MOF for eCO_2RR

Catalyst and Synthesis	Cell System	Electrolyte	Overpotential [V vs. RHE]	FE	Stability	Ref.
Oxide-derived Cu/C microcrystals prepared by pyrolyzing HKUST-1 under Ar	H-cell	0.1 м $KHCO_3$	−0.5	34.8% for C_2H_5OH	-	[5]
Cu clusters formed by calcining HKUST-1	Flow cell	1 м KOH	−1.07	45% for C_2H_4	-	[6]
Cu_2O/CuO microcrystals prepared by calcining HKUST-1	H-cell	0.1 м $KHCO_3$	−1.58	51.0% C_2H_4 70.0% for C_{2+}	20 h	[7]
	Flow cell	1 м KOH	at −320 mA cm^{-2}	49.8% for C_2H_4 72.0% for C_{2+}	-	
10 wt% PTFE mixed $Cu_xO_yC_z$ obtained by pyrolyzing HKUST-1	Flow cell	1 м KOH	at −80 mA cm^{-2}	54% for C_{2+}	-	[8]
Defect-engineered HKUST-1 with coordinatively unsaturated Cu paddle wheel clusters	H-cell	0.1 м $KHCO_3$	−0.80	36% for C_2H_4	5 h	[9]
**n-MDC-250 prepared by calcining Cu-MOF nanoparticles**	**H-cell**	**0.1 м $KHCO_3$**	**−1.01**	**63.1% for C_2H_4 80.9% for C_{2+}**	**10 h**	**This work**
	**Flow cell**	**1 м KOH**	**−0.56**	**45.6% for C_2H_4 70.6% for C_{2+}**	**2 h**	**This work**

Table S12. Comparison of oxide-derived copper catalysts for eCO_2RR

Catalyst	Cell System	Electrolyte	Overpotential [V vs. RHE]	FE	Stability	Ref.
O_2 plasma treated Cu film	H-cell	0.1 м $KHCO_3$	−0.9	60% for C_2H_4	5 h	[10]
O_2 plasma treated Cu nanocubes	H-cell	0.1 м $KHCO_3$	−1.0	45% for C_2H_4<br>73% for C_{2+}	-	[11]
Electrochemically fragmented Cu_2O nanoparticles	H-cell	0.1 м $KHCO_3$	−1.1	57.3% for C_2H_4<br>74.0% for C_{2+}	-	[12]
Branched CuO nanoparticles acquired by selective oxidation of Cu_2O nanocubes	H-cell	0.1 м $KHCO_3$	−1.05	70% for C_2H_4	12 h	[13]
Oxygen vacancy rich CuO nanodendrites formed by controlled electroreduction	H-cell	0.1 м $KHCO_3$	−1.4	63% for C_2H_4	9 h	[14]
Solvothermally synthesized truncated-octahedral Cu_2O nanoparticles	H-cell	0.5 м $KHCO_3$	−1.1	59% for C_2H_4	2 h	[15]
Multihollow Cu_2O nanoparticles acquired by acidic corrosion	Flow cell	2 м KOH	−0.61	75.2% for C_{2+}	3 h	[16]
Rapidly cooled CuO nanoparticles	Flow cell	1 м KOH	−1.05	31.2% for C_2H_4<br>74.0% for C_{2+}	-	[17]
Poly(vinylidene fluoride) modified CuO nanoparticles	H-cell	0.5 м $KHCO_3$	−1.22	40.6% for C_2H_4	6 h	[18]
Hydrothermally synthesized octahedral Cu_2O nanostructures	H-cell	0.5 м $KHCO_3$	−0.3	30.5% for C_{2+} alcohols	1.2 h	[19]
**n-MDC-250 prepared by calcining Cu-MOF nanoparticles**	**H-cell**	**0.1 м $KHCO_3$**	−1.01	**63.1% for C_2H_4**<br>**80.9% for C_{2+}**	**10 h**	**This work**
	**Flow cell**	**1 м KOH**	−0.56	**45.6% for C_2H_4**<br>**70.6% for C_{2+}**	**2 h**	**This work**

Table S13. Partial current density of the products normalized by ECSA (j_{ECSA}) at −1.01 V vs. RHE in neutral condition.

Products	**n-MDC-250**	**m-MDC-265**
Hydrogen	−2.65 mA cm^{-2}	−6.25 mA cm^{-2}
C_1 Products	−1.51 mA cm^{-2}	−1.89 mA cm^{-2}
C_{2+} Products	−16.9 mA cm^{-2}	−13.6 mA cm^{-2}
Total	−20.6 mA cm^{-2}	−21.7 mA cm^{-2}

Table S14. Cu K-edge XANES linear combination fitting results of n-MDC-250 and m-MDC-265 before and after electrochemical activation

Sample	Status	Cu [%]	Cu_2O [%]	CuO [%]
n-MDC-250	Before the activation	0	0	100
	After the activation	61.7	28.8	9.5
	in-situ at −1.0 V vs. RHE	76.5	23.5	0
m-MDC-265	Before the activation	0	0	100
	After the activation	61.0	37.6	1.4
	in-situ at −1.0 V vs. RHE	75.6	24.4	0

The linear combination fittings for XANES were implemented for the energy region between 8970 eV and 9000 eV.

Table S15. Summary of structural parameters of n-MDC-250 and m-MDC-265 before activation acquired from Cu K-edge EXAFS fitting

Sample	Shell	CN[a)]	R [nm][b)]	σ^2 [nm^2][c)]	R-factor
n-MDC-250 before activation	$Cu–O_1$	3.25 ± 0.242	0.196 ± 0.000	32.3 ± 9.4	0.00789
	$Cu–O_2$	0.647 ± 0405	0.278 ± 0.001	32.3 ± 9.4	
	$Cu–Cu_1$	3.65 ± 1.10	0.291 ± 0.001	73.2 ± 27.0	
	$Cu–Cu_2$	3.65 ± 1.10	0.307 ± 0.001	73.2 ± 27.0	
	$Cu–Cu_3$	1.82 ± 0.549	0.322 ± 0.005	73.2 ± 27.0	
m-MDC-265 before activation	$Cu–O_1$	3.14 ± 0.323	0.196 ± 0.000	35.1 ± 12.6	0.00977
	$Cu–O_2$	0.802 ± 0.671	0.276 ± 0.003	35.1 ± 12.6	
	$Cu–Cu_1$	3.88 ± 1.91	0.291 ± 0.001	80.3 ± 46.9	
	$Cu–Cu_2$	3.88 ± 1.91	0.307 ± 0.001	80.3 ± 46.9	
	$Cu–Cu_3$	1.94 ± 0.956	0.320 ± 0.004	80.3 ± 46.9	

[a)] CN refers to the coordination number; [b)] R represents the interatomic distance.; [c)] σ^2 describes the Debye-Waller factor. The fitting ranges were set to be $1.10 \le R$ (Å) ≤ 3.10. The single scattering pathways were obtained from the FEFF calculation of the CuO crystalline structure.

Table S16. Summary of structural parameters of n-MDC-250 and m-MDC-265 after activation acquired from Cu K-edge EXAFS fitting

Sample	Shell	CN[a)]	R [nm][b)]	σ^2 [nm^2][c)]	R-factor
n-MDC-250 after activation	Cu–O_1 (Cu_2O)	1.35	0.188 ± 0.000	21.3	0.00596
	Cu–Cu (Cu)	7.00 ± 0.629	0.257 ± 0.001	84.6 ± 7.8	
	Cu–Cu (Cu_2O)	3.55	0.274 ± 0.001	123.5 ± 29.8	
m-MDC-265 after activation	Cu–O_1 (Cu_2O)	1.31	0.185 ± 0.001	24.3	0.00649
	Cu–Cu (Cu)	7.40 ± 0.426	0.253 ± 0.001	56.5 ± 5.1	
	Cu–Cu (Cu_2O)	4.06	0.262 ± 0.004	31.6 ± 5.5	

[a)] CN refers to the coordination number; [b)] R represents the interatomic distance; [c)] σ^2 describes the Debye-Waller factor. The fitting ranges were set to be $1.05 \leq R$ (Å) ≤ 2.95. The single scattering pathways were obtained from the FEFF calculation of Cu and Cu_2O crystalline structure.

Supplementary References

[1] S. Nakayama, T. Kaji, M. Shibata, T. Notoya, T. Osakai, *J. Electrochem. Soc.* **2007**, *154*, C1.

[2] H. Zhang, Y. Zhang, Y. Li, S. Ahn, G. T. R. Palmore, J. Fu, A. A. Peterson, S. Sun, *Nanoscale* **2019**, *11*, 12075.

[3] L. Spillane, P. Longo, B. Schaffer, B. Miller, P. Thomas, R. Twesten, *Microsc. Microanal.* **2020**, *26*, 1676.

[4] Q. Lei, H. Zhu, K. Song, N. Wei, L. Liu, D. Zhang, J. Yin, X. Dong, K. Yao, N. Wang, X. Li, B. Davaasuren, J. Wang, Y. Han, *J. Am. Chem. Soc.* **2020**, *142*, 4213.

[5] K. Zhao, Y. Liu, X. Quan, S. Chen, H. Yu, *ACS Appl. Mater. Interfaces* **2017**, *9*, 5302.

[6] D.-H. Nam, O. S. Bushuyev, J. Li, P. De Luna, A. Seifitokaldani, C.-T. Dinh, F. P. García de Arquer, Y. Wang, Z. Liang, A. H. Proppe, C. S. Tan, P. Todorović, O. Shekhah, C. M. Gabardo, J. W. Jo, J. Choi, M.-J. Choi, S.-W. Baek, J. Kim, D. Sinton, S. O. Kelley, M. Eddaoudi, E. H. Sargent, *J. Am. Chem. Soc.* **2018**, *140*, 11378.

[7] K. Yao, Y. Xia, J. Li, N. Wang, J. Han, C. Gao, M. Han, G. Shen, Y. Liu, A. Seifitokaldani, X. Sun, H. Liang, *J. Mater. Chem. A* **2020**, *8*, 11117.

[8] N. Sikdar, J. R. C. Junqueira, S. Dieckhöfer, T. Quast, M. Braun, Y. Song, H. B. Aiyappa, S. Seisel, J. Weidner, D. Öhl, C. Andronescu, W. Schuhmann, *Angew. Chem. Int. Ed.* **2021**, *60*, 23427.

[9] W. Zhang, C. Huang, J. Zhu, Q. Zhou, R. Yu, Y. Wang, P. An, J. Zhang, M. Qiu, L. Zhou, L. Mai, Z. Yi, Y. Yu, *Angew. Chem. Int. Ed.* **2022**, *61*, e202112116.

[10] H. Mistry, A. S. Varela, C. S. Bonifacio, I. Zegkinoglou, I. Sinev, Y.-W. Choi, K. Kisslinger, E. A. Stach, J. C. Yang, P. Strasser, B. R. Cuenya, *Nat. Commun.* **2016**, *7*, 12123.

[11] D. Gao, I. Zegkinoglou, N. J. Divins, F. Scholten, I. Sinev, P. Grosse, B. R. Cuenya, *ACS Nano* **2017**, *11*, 4825.

[12] H. Jung, S. Y. Lee, C. W. Lee, M. K. Cho, D. H. Won, C. Kim, H.-S. Oh, B. K. Min, Y. J. Hwang, *J. Am. Chem. Soc.* **2019**, *141*, 4624.

[13] J. Kim, W. Choi, J. W. Park, C. Kim, M. Kim, H. Song, *J. Am. Chem. Soc.* **2019**, *141*, 6986.

[14] Z. Gu, N. Yang, P. Han, M. Kuang, B. Mei, Z. Jiang, J. Zhong, L. Li, G. Zheng, *Small Methods* **2019**, *3*, 1800449.

[15] Y. Gao, Q. Wu, X. Liang, Z. Wang, Z. Zheng, P. Wang, Y. Liu, Y. Dai, M.-H. Whangbo, B. Huang, *Adv. Sci.* **2020**, *7*, 1902820.

[16] P.-P. Yang, X.-L. Zhang, F.-Y. Gao, Y.-R. Zheng, Z.-Z. Niu, X. Yu, R. Liu, Z.-Z. Wu, S. Qin, L.-P. Chi, Y. Duan, T. Ma, X.-S. Zheng, J.-F. Zhu, H.-J. Wang, M.-R. Gao, S.-H. Yu, *J. Am. Chem. Soc.* **2020**, *142*, 6400.

[17] C. Yang, H. Shen, A. Guan, J. Liu, T. Li, Y. Ji, A. M. Al-Enizi, L. Zhang, L. Qian, G. Zheng, *J. Colloid Interface Sci.* **2020**, *570*, 375.

[18] H.-Q. Liang, S. Zhao, X.-M. Hu, M. Ceccato, T. Skrydstrup, K. Daasbjerg, *ACS Catal.* **2021**, *11*, 958.

[19] B. Liu, X. Yao, Z. Zhang, C. Li, J. Zhang, P. Wang, J. Zhao, Y. Guo, J. Sun, C. Zhao, *ACS Appl. Mater. Interfaces* **2021**, *13*, 39165.
